# Benzene, Toluene, and Monosubstituted Derivatives: Diabatic Nature of the Oscillator Strengths of S_1_ ← S_0_ Transitions

**DOI:** 10.1021/acs.jpca.1c01685

**Published:** 2021-06-16

**Authors:** David Robinson, Saleh S. Alarfaji, Jonathan D. Hirst

**Affiliations:** † Department of Chemistry and Forensics, School of Science and Technology, Nottingham Trent University, Clifton Lane, Nottingham NG11 8NS, United Kingdom; ‡ School of Chemistry, University of Nottingham, University Park, Nottingham NG7 2RD, United Kingdom

## Abstract

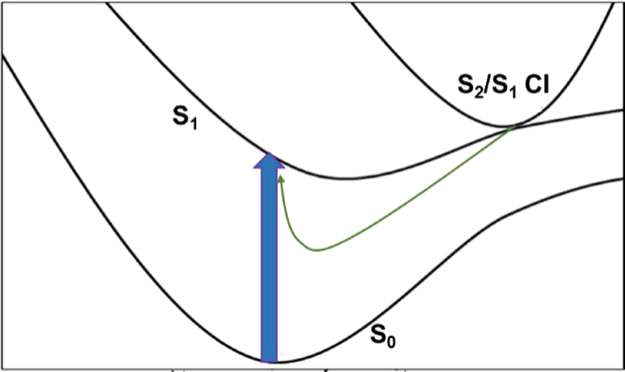

For benzene, toluene, aniline, fluorobenzene, and phenol, even sophisticated treatments of electron correlation, such as MRCI and XMS-CASPT2 calculations, show oscillator strengths typically lower than experiment. Inclusion of a simple pseudo-diabatization approach to perturb the S_1_ state with approximate vibronic coupling to the S_2_ state for each molecule results in more accurate oscillator strengths. Their absolute values agree better with experiment for all molecules except aniline. When the coupling between the S_1_ and S_2_ states is strong at the S_0_ geometry, the simple diabatization scheme performs less well with respect to the oscillator strengths relative to the adiabatic values. However, we expect the scheme to be useful in many cases where the coupling is weak to moderate (where the maximum component of the coupling has a magnitude less than 1.5 au). Such calculations give an insight into the effects of vibronic coupling of excited states on UV/vis spectra.

## Introduction

Small monosubstituted benzenes serve as model systems for biological chromophores, helping to understand the structure of proteins^1^ and hydrogels.^2^ Both their electronically excited states^3^ and their vibrational spectra have been widely investigated. For example, the aromatic groups of tyrosine and phenylalanine contribute to the electronic circular dichroism of proteins in the near ultraviolet,^4^ while IR spectroscopy is widely used to probe the conformational landscape of proteins. Toluene plays a role in atmospheric chemistry, oxidizing in the troposphere and playing a role in secondary organic aerosol formation.^5−8^ Toluene is also important for the synthesis of industrial polymers,^9^ and excited states have a key role in the radiolysis of aromatic compounds.^10^ A comprehensive description of the spectroscopy of individual chromophores is a pre-requisite for understanding the often complex spectra of dimers^11^ and higher aggregates present in many types of macromolecular systems. We have a long-standing interest in the accurate and efficient description of the spectroscopy of toluene as a model of phenylalanine for electronic circular dichroism calculations. Such calculations determine parameters for our DichroCalc software.^12,13^ In particular, we are interested in a simple, efficient, and quantitative approach to the calculation of vibronic coupling of different electronically excited states in such molecules to improve the fine structure of the electronic transitions and corresponding transition dipole moments.

To glean useful information from calculations of the electronic excited states of benzene and monosubstituted benzene derivatives, one must understand the nature of the transitions being studied: in our case, the S_1_ ← S_0_ transition. In benzene, the S_1_ ← S_0_ (Ã^1^B_2u_ ← X̃^1^A_1g_) transition is formally forbidden, but it becomes allowed because of vibronic coupling to the optically allowed C̃^1^E_1u_ state.^14,15^ Monosubstituted halobenzenes have *C*_2*v*_ symmetry, and so the S_1_ ← S_0_ transition becomes formally allowed, exhibiting a larger oscillator strength than benzene, although still weak. This is often stated as the electronic structure of monosubstituted benzenes having a “memory” of the *D*_6*h*_ symmetry and vibronic nature of the transition. Experimental studies have consistently shown some intensity, with activity in the *b*_2_ vibrational modes in the S_1_ ← S_0_ spectra.^16^ The S_2_ state is known to have a conical intersection, leading to fast internal conversion to the S_1_ state, with the S_2_ state having a lifetime of less than 100 fs.^17,18^ Once on the S_1_ surface, the excitation wavepacket is able to decay along two channels: the first to the nearby S_1_/S_0_ conical intersection and the second to the S_1_ minimum.^19^ The S_1_ state is longer lived, with a lifetime of ∼4 ps.^20^

There have been several different computational approaches to the accurate description of S_1_ vibrational frequencies of aromatic molecules and vibronic coupling of S_1_ states to higher electronic states for benzene, toluene, and other monosubstituted benzene derivatives. The vibronic bands in benzene have been investigated using multireference approaches,^21^ and coupling between different states^22^ has been considered in the interpretation of the photochemistry observed experimentally (see also ref (23) for a useful review by Suzuki). Tew et al. investigated the anharmonic nature of the S_1_ vibrational frequencies of toluene using the CC2/cc-pVTZ approach.^24^ They found several modes with substantial anharmonicity, and their overall agreement with experiment was within 30 cm^–1^ for all vibrational modes. Wang et al. studied the quantum dynamics of aniline, discovering vibronic coupling between the S_1_ state and two Rydberg states.^25^ Lykhin et al. also showed the importance of triplet states in the photodynamics of aniline, with a competitive photorelaxation route from the ^1^ππ* state.^26^ Mondal and Mahapatra determined that the S_1_ state of fluorobenzene was coupled to a manifold of higher singlet excited states by constructing a vibronic Hamiltonian based on EOM-CCSD calculations.^27,28^ Phenol exhibits vibronic coupling between the S_1_ state and the dissociative S_2_ state of a πσ* character.^29^ Much theoretical work has been performed, confirming the nature of this conical intersection and tunneling, which is also part of the photodissociation pathway.^30−33^ While each of these approaches shows good qualitative and quantitative accuracy in the low energy transitions for these molecules, they require specialist work and attention crafted for each individual molecule and are not applicable in an “off-the-shelf” sense, accessible to users from different disciplines.

In the current work, we investigate the S_1_ ← S_0_ transition in toluene. We employ a simple diabatization scheme to include vibronic coupling effects approximately. This scheme is applied to benzene and four monosubstituted derivatives to explore oscillator strength enhancement from vibronic coupling for multireference CI (MRCI) and XMS-CASPT2 calculations that is amenable to non-specialist users.

## Computational Details

**Figure 1:**
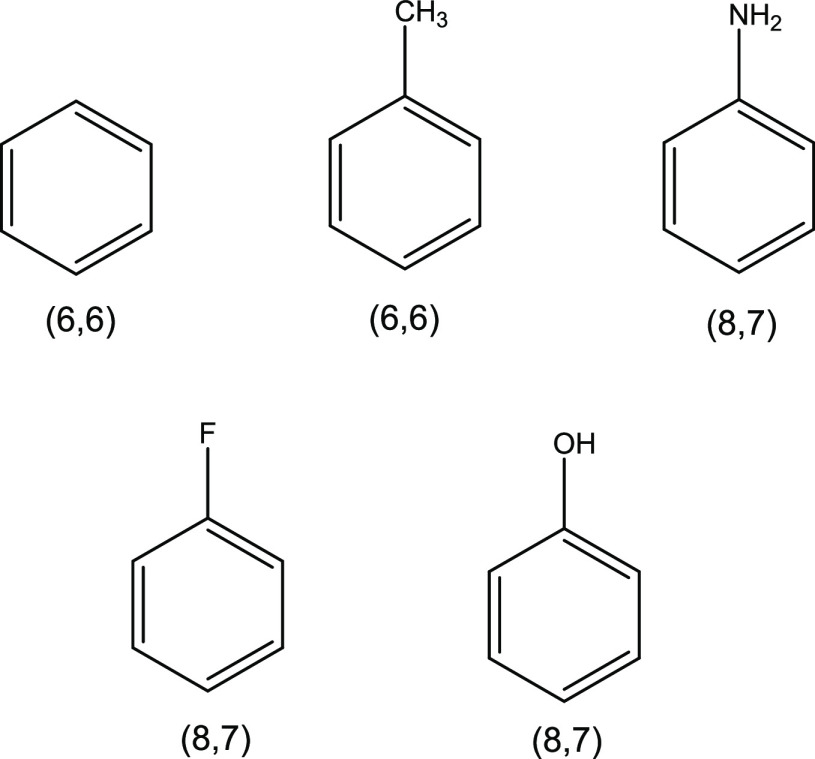
Benzene and the monosubstituted benzene derivatives investigated in this work. CASSCF active spaces are given in parentheses, where the notation is (number of active electrons, number of active orbitals).

The S_0_ and S_1_ equilibrium geometries and S_2_/S_1_ minimum energy conical intersection (MECI) geometry for each of the molecules in Figure 1 were calculated at the XMS-CASPT2/cc-pVTZ level of theory (active spaces shown in Figure 1; in each case, the π-electron system plus lone pairs were included).

**Figure 2:**
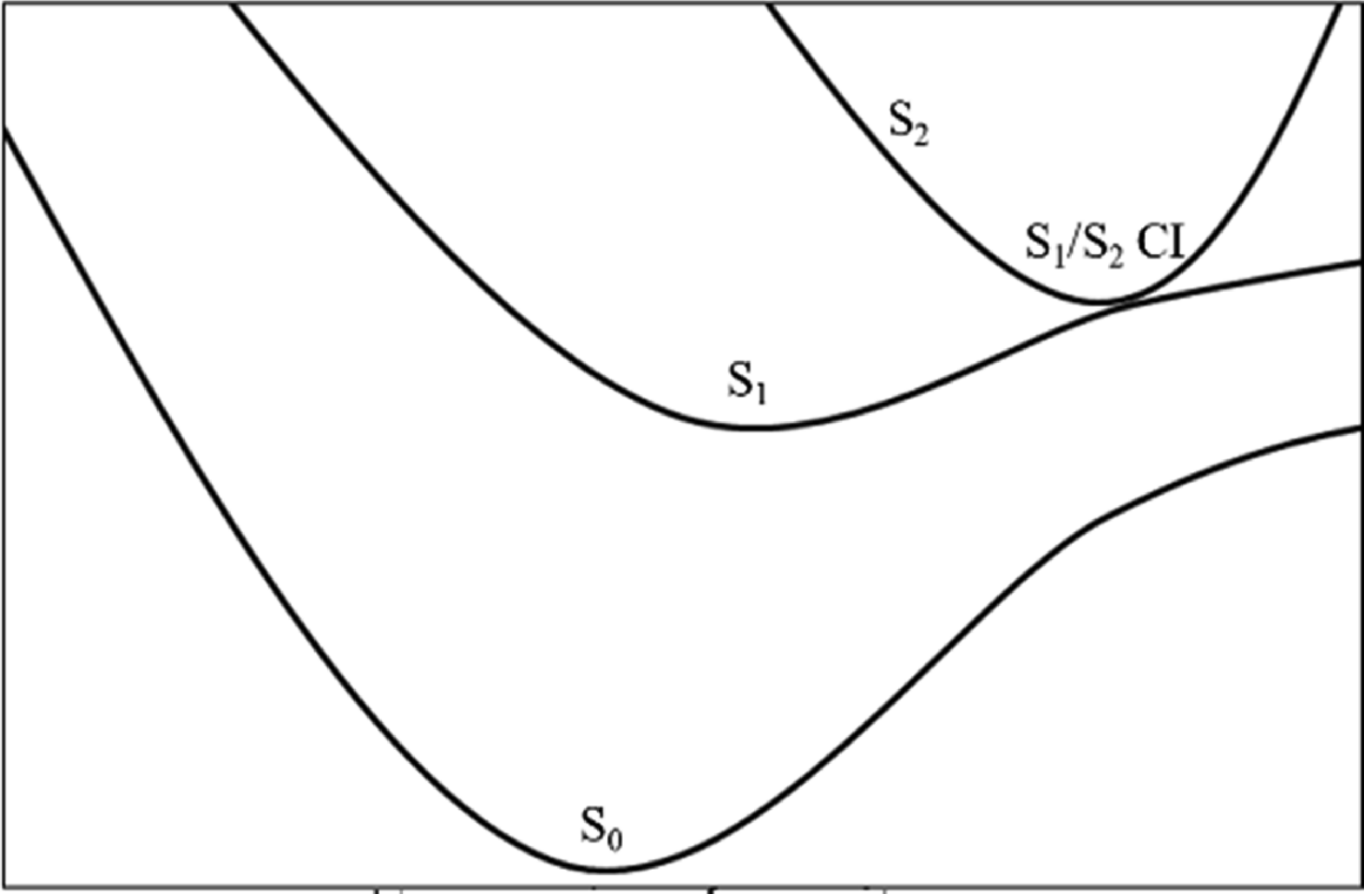
Qualitative schematic of the S_0_, S_1_, and S_2_ potential energy surfaces in the region of the Franck–Condon excitation.

Vibronic coupling is a process where the Born–Oppenheimer approximation breaks down and an adiabatic electronic state, *J*, mixes with another adiabatic electronic state, *I*, due to vibrations of the nuclei:
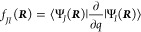
1where *f_JI_* are the non-adiabatic coupling matrix elements (NACMEs) and ***R*** are the nuclear coordinates. The effects of vibronic coupling were included using the simple diabatization scheme of Simah et al.^34^ (based on the work by Domcke and Woywod^35^), in which the overlap of the orbitals from a reference geometry and target geometry is optimized and the resulting pseudo-diabatic orbitals are used to transform the wavefunction at the target geometry. In our case, we chose the reference geometry to be the MECI of the S_2_/S_1_ conical intersection seam, as this is the point at which the two states involved in the intensity borrowing process interact most strongly. The target geometry is the S_0_ optimized geometry as this represents the geometry at which the Franck–Condon (FC) excitation occurs. The diabatic states (denoted by the superscript *d*) are considered to be a minor perturbation to the adiabatic states and are found by a unitary transformation of the S_1_ and S_2_ adiabatic states (denoted by a superscript *a*)
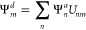
2The unitary transformation matrix is chosen such that the NACME vector, *X*_2_
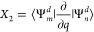
3is minimized for all of the internal coordinates, *q*. For a two-state diabatization, the unitary transformation matrix, **U**, is given as
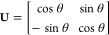
4where a single non-adiabatic mixing angle, θ, can be used to describe the mixing of the adiabatic states. In the approximate scheme used in this work, the CI coefficients from an MRCI or XMS-CASPT2 calculation were transformed by maximizing the overlap of the CASSCF orbitals at the S_0_ geometry with those obtained at a reference geometry, generating a pseudo-diabatic set of orbitals:
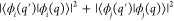
5where the overlap is computed over all active orbitals *i* and *j* at the current geometry *q* with those at the reference geometry *q*′, which in this case was the S_2_/S_1_ MECI. In all cases, we assume that this MECI lies close to the S_1_ minimum and the proximity of the electronic states allows them to interact (see Figure 2 for a qualitative overview). The diabatic wavefunction, Ψ_*m*_^*d*^, is constructed from the pseudo-diabatic orbitals as
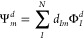
6At the target geometry, the matrix **d** is related to the adiabatic wavefunctions by the transformation **d = cU**, where **c** is the coefficient matrix of the adiabatic wavefunctions and **U** is determined using the condition that **d** remains as close as possible to the matrix **d^ref^** at the reference geometry:
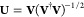
7where
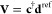
8The transition dipole moments can then be calculated for the S_1_ ← S_0_ transition, with the approximately diabatic S_1_ state, as
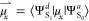
9and similarly for the *_y_* and *_z_* components using either the MRCI or XMS-CASPT2 computed densities. Writing the energy expressions explicitly for each of the two states, one obtains
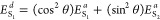
10a
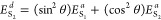
10bThe oscillator strength can then be calculated:
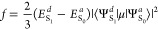
11While in eq 11, we use an adiabatic description of the S_0_ state and pseudo-diabatic representation for S_1_, the pseudo-diabatic representation is essentially only a perturbation to the adiabatic S_1_ state. As such, where there is very strong coupling between S_1_ and S_2_ states, we expect this simple approximation to break down as the pseudo-diabatization scheme is based on the assumption that the orbitals and CI coefficients change very little as a function of geometry; this is not always true in the vicinity of a conical intersection. In the original scheme of Simah et al.,^34^ the reference geometry is ideally chosen where the adiabatic and diabatic states are identical (e.g., due to symmetry). In the current work, the use of the S_2_/S_1_ MECI is a point at which the NACME terms do not vanish completely, but the adiabatic and diabatic states may not be identical. Additionally, the reference orbitals at the MECI geometry may have poor overlap with those at the target geometry (S_0_). If the MECI is far from the FC region of the S_1_ state, then the current scheme is likely to show limited vibronic coupling, even if there is true coupling between the two states.

Adiabatic XMS-CASPT2^36^ calculations were performed within the single-state single-reference contraction scheme (SS-SR) and a real shift of 0.2 au, using the cc-pVTZ basis^37^ and the cc-pVTZ-JKFIT auxiliary basis set,^38^ using the BAGEL software.^39,40^ Adiabatic time-dependent density functional theory (TDDFT) calculations within the Tamm–Dancoff approximation^41^ were performed with the B3LYP,^42^ CAM-B3LYP,^43^ M06-2X,^44^ and ωB97X^45^ functionals. Single-reference EOM-CCSD,^46^ ADC(2),^47^ and ADC(3)^48^ calculations were also performed. TDDFT and single-reference wavefunction theory calculations were performed using the Q-Chem software.^49^ The diabatic transformation calculations (using both internally contracted MRCI^50−52^ and XMS-CASPT2) were performed with the Molpro software suite.^53^ The S_0_ and S_2_/S_1_ calculated geometries were superposed based on minimizing the RSMD of all atoms. In all cases, the cc-pVTZ basis set^37^ was employed as it represents a good compromise between accuracy and computational cost.

In addition, for toluene, a vibrationally resolved spectrum was determined by calculating the FC factors between the S_0_ and S_1_ harmonic vibrational modes and frequencies. The spectrum was calculated using the ezSpectrum software^54,55^ at a temperature of 10 K.

## Results and Discussion

**Figure 3:**
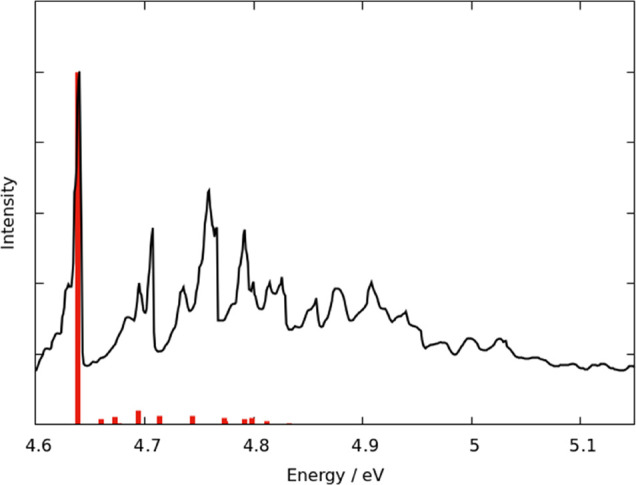
Experimental (line) and computed (stick) spectrum of the S_1_ ← S_0_ transition for toluene. The computed spectrum has been shifted by −0.136 eV to match the experimental spectrum.^60^

We first consider the S_0_ and S_1_ states of toluene. In Table 1 are the calculated XMS-CASPT2 harmonic vibrational frequencies. The scaled harmonic vibrational frequencies show fair agreement with experiment,^16,56−58^ with a maximum error of 316 cm^–1^ for one of the low frequency carbon–carbon bend modes (m_18_) and average errors of 55 and 29 cm^–1^ for the S_0_ and S_1_ frequencies, respectively, after scaling. The average error for the S_0_ vibrations is 45 cm^–1^, neglecting the m_18_ frequency. Tew et al. employed the CC2/cc-pVTZ approach to calculate anharmonic frequencies of toluene.^24^ The differences exhibited between the XMS-CASPT2 and experimental S_1_ frequencies are likely due to a combination of anharmonicity, for which CC2/cc-pVTZ performs well,^24^ and potential issues in the XMS-CASPT2 accuracy. In particular, the m_4_, m_12_, m_15_, m_16_, m_18_, m_23_, and m_25_ modes all show larger differences to the CC2 values (and experiment); these were modes identified as genuinely anharmonic.^24^ Battaglia and Lindh determined XMS-CASPT2 excitations to be poor relative to MS-CASPT2 in regions where potential surfaces are energetically well separated (i.e., at or near minima); they developed an alternative approach to XMS-CASPT2 termed extended dynamically weighted CASPT2 (XDW-CASPT2).^59^ The results presented here suggest that stationary points and their frequencies may be similarly affected. These frequencies have been used to generate a vibrationally resolved spectrum (Figure 3). The dominant transition is the 0–0 vibrational line, with a handful of other vibrational lines about two orders of magnitude smaller.

**Table 1:** Calculated Harmonic Frequencies of the S_0_ and S_1_ States of Toluene (XMS-CASPT2/cc-pVTZ)^c^

	S_0_		S_1_	
assignment^a^	XMS-CASPT2	Expt.^b^	XMS-CASPT2	Expt.^b^
m_1_	3072	3087	3086	3097
m_2_	3052	3063	3076	3077
m_3_	3038	3055	3066	3063
m_4_	1560	1605	1411	
m_5_	1439	1494	1401	
m_6_	1179	1210	1162	1193
m_7_	1136	1175	1110	1021
m_8_	1003	1030	921	935; 934
m_9_	949	1003	904	966
m_10_	751	785	719	736; 753
m_11_	492	521	435	457
m_12_	798	964	514	687
m_13_	751	843	511	
m_14_	379	407	211	228; 226
m_15_	798	978	583	
m_16_	751	895	514	697
m_17_	637	728	511	
m_18_	379	695	309	423
m_19_	317	464	287	320; 314
m_20_	197	216	131	157; 145
m_21_	3058	3039	3086	3087
m_22_	3038	3029	3066	3048
m_23_	1560	1586	1528	
m_24_	1424	1445	1411	
m_25_	1340	1312	1331	
m_26_	1277	1280	1248	
m_27_	1136	1155	1110	
m_28_	1049	1080	1000	
m_29_	587	623	514	532
m_30_	317	342	309	332; 331

a Assignments taken from ref (14).

b Experimental data taken from refs (16, 56, 58).

c Harmonic frequencies are scaled by 0.954. See the Supporting Information for full details of the scaling parameter.

**Figure 4:**
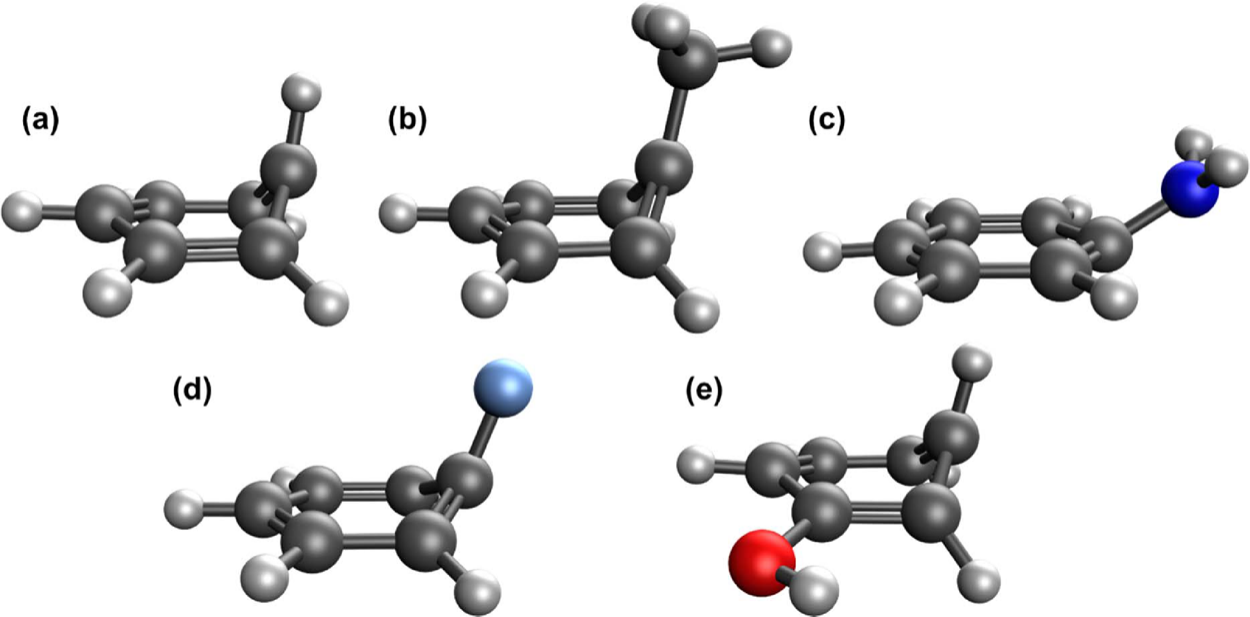
XMS-CASPT2/cc-pVTZ structures for the S_2_/S_1_ MECI of (a) benzene, (b) toluene, (c) aniline, (d) fluorobenzene, and (e) phenol.

We now turn to the calculation of the oscillator strengths for the S_1_ ← S_0_ transition for toluene, benzene, and three monosubstituted benzene derivatives. The S_2_/S_1_ MECI structures for each of the molecules considered are shown in Figure 4. With the exception of aniline, all exhibit a prefulvene-like structure typical of the MECI geometries of aromatic molecules. Aniline exhibits geometrical distortion of the −NH_2_ group relative to the ring, with the atoms in the ring remaining planar. This is similar to that seen for the ^1^ππ*/^1^πσ* MECI in the recent work of Ray and Ramesh.^61^ The MECI geometry for toluene has a peaked topology, while the rest have a sloped topology.

**Figure 5:**
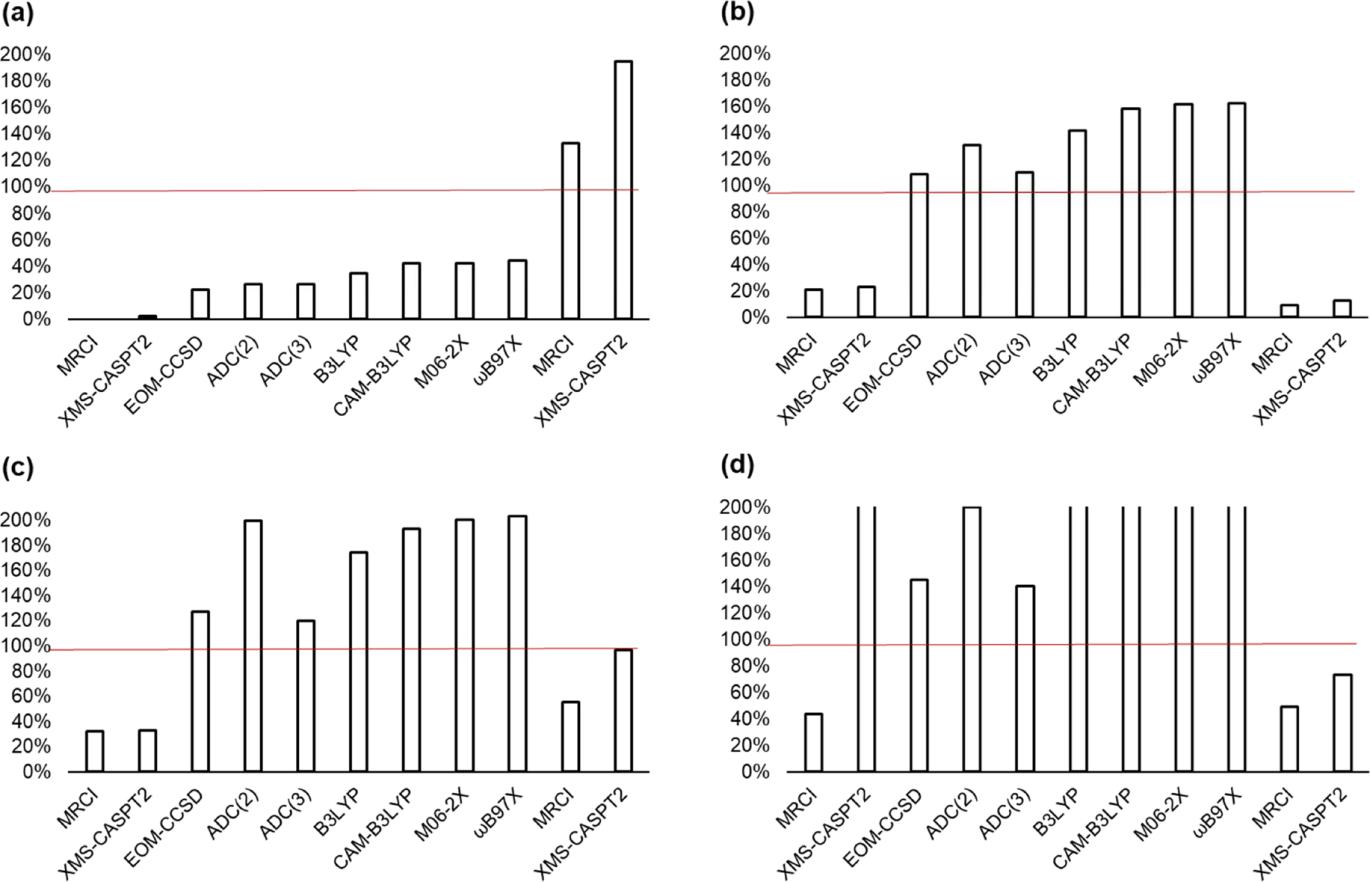
Calculated oscillator strengths expressed as a percentage of the experimental value. A value of 100% corresponds to the experimental value. The final two columns of each plot are the pseudo-diabatic MRCI and XMS-CASPT2 oscillator strengths. (a) Toluene; (b) aniline; (c) fluorobenzene; and (d) phenol. Values greater than 200% are depicted with open boxes.

**Figure 6:**
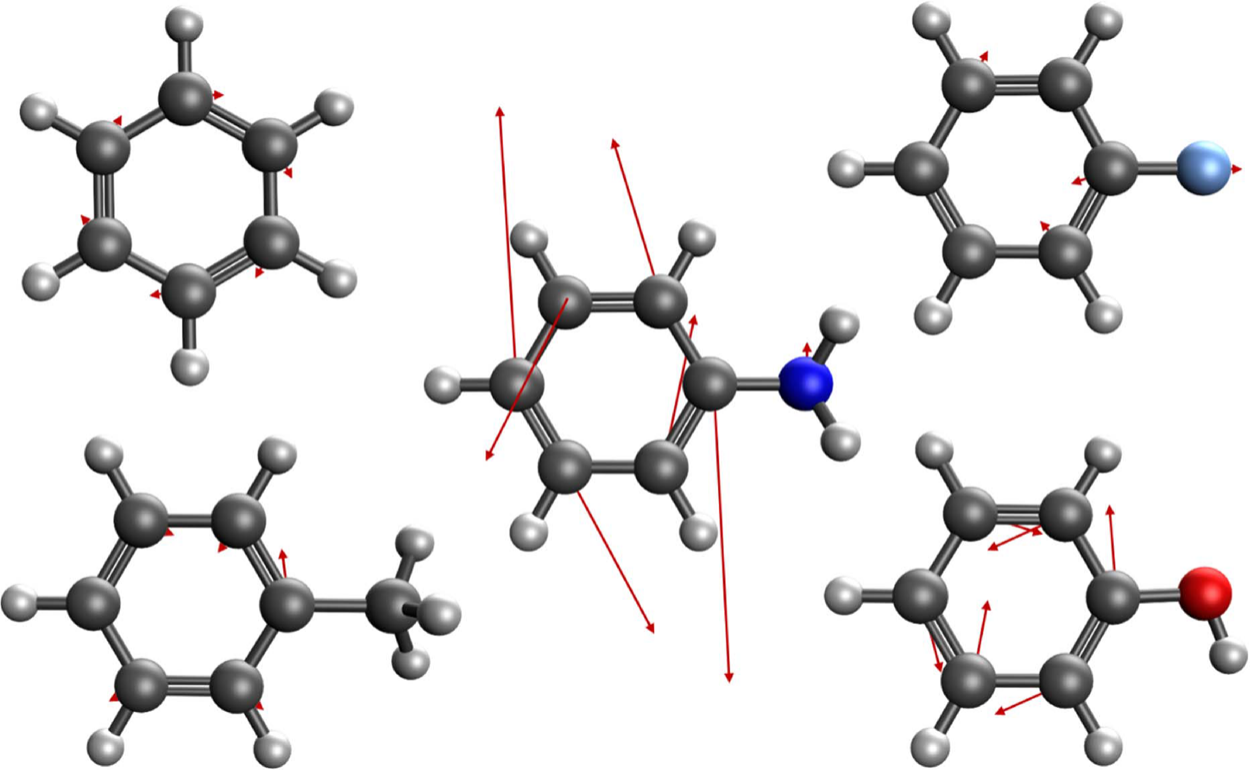
Visual representation of the non-adiabatic coupling vectors between the S_2_ and S_1_ states at the S_0_ optimized geometries for benzene (top left), toluene (bottom left), aniline (center), fluorobenzene (top right), and phenol (bottom right).

The computed transition energies are given in Table 2 (0–0 transitions) and Table 3 (Franck–Condon transitions), along with the calculated oscillator strengths. The MECIs lie 1.14, 0.89, 0.52, 0.59, and 1.10 eV above the S_1_ minima and 0.97, 0.73, 0.28, 0.42, and 1.03 eV above the Franck–Condon transition energy (S_1_ ← S_0_) for benzene, toluene, aniline, fluorobenzene, and phenol, respectively. The magnitudes of the calculated and experimental oscillator strengths^62^ are compared in Figure 5. The single-reference methods generally overestimate the oscillator strength, although for benzene (data shown in Table 3) and toluene, they are between 0 and 50% of the experimental value. The multireference methods both underestimate the oscillator strengths in comparison to experiment and the single-reference methods (DFT, EOM-CCSD, and ADC approaches), with the exception of phenol, where the XMS-CASPT2 oscillator strength is the largest of all the methods considered. The pseudo-diabatic oscillator strengths are given in Table 3 and Figure 5 for MRCI and XMS-CASPT2. The calculated oscillator strengths are enhanced relative to the adiabatic values for all molecules except aniline, where the pseudo-diabatic values are ∼50% of the adiabatic values and ∼10% of the experimental value for both MRCI and XMS-CASPT2. In this case, we can see that the S_2_ state is energetically close to the S_1_ state across the potential energy surface connecting the S_0_ minimum and S_2_/S_1_ conical intersection (see Figure S1), deviating by no more than ∼1.1 eV. In contrast, the other molecules have energy gaps greater than 1.5 eV at the S_0_ minima. In Figure 6, we present visual representations of the XMS-CASPT2 calculated non-adiabatic coupling vector between the S_2_ and S_1_ states at the S_0_ geometry. It is clear for aniline that the coupling is much stronger than that seen for the other molecules. This is also reflected in the Franck–Condon excitation energy being less than 0.3 eV lower than the S_2_/S_1_ MECI relative to the S_0_ energy. Interestingly, the coupling is strongest for the atoms in the ring and relatively low for the −NH_2_ group, in contrast to the ^1^ππ*/^1^πσ* conical intersection.^61^ Worth and co-workers demonstrated two 3p Rydberg states between the S_1_ and S_2_ states. These also couple to the S_1_ state,^25^ but they are not considered in the current study. We propose that, in this case, the approximate diabatization scheme would need to be replaced with a more robust approach (possibly including Franck–Condon factors and explicit integration of the NACMEs) to give a more accurate oscillator strength as vibronic coupling between the S_1_ and S_2_ states is stronger than the other molecules considered. Given in Figure S2 are the maximum and average coupling values compared to the difference in oscillator strength between the calculated and experimental oscillator strengths. For the molecules considered, the accuracy of the current method deteriorates when an individual atom’s NACME vector has a magnitude greater than 1.5 au (or the average magnitude of the NACME vector across all atoms is greater than ∼0.7 au). The coupling between electronically excited states for phenol in this study is between two ^1^ππ* states, while the true S_2_ state is of a πσ* character.^63^ This is a consequence of the approach taken in this study, namely, choosing the simple π-electron active space and not expanding to include σ* orbitals.

**Table 2:** Calculated Energy Differences (XMS-CASPT2/cc-pVTZ) between the Minima for the S_0_ and S_1_ States of Each Molecule and Their S_2_S_1_ MECIs

molecule	Δ*E* (0–0, S_1_ ← S_0_) (eV)	Δ*E* (S_2_/S_1_ ← S_0_) (eV)
benzene	4.72	5.86
toluene	4.60	5.49
aniline	4.29	4.81
fluorobenzene	4.69	5.28
phenol	4.53	5.63

**Table 3:** Computed Franck–Condon Excitation Energies (in eV) and Oscillator Strengths in the Adiabatic and Pseudo-diabatic Basis^a^

	benzene		toluene		aniline		fluorobenzene		phenol	
method	Δ*E*	*f*	Δ*E*	*f*	Δ*E*	*f*	Δ*E*	*f*	Δ*E*	*f*
Adiabatic										
MRCI	5.08	0.0000	4.98	0.0000	4.83	0.0074	5.08	0.0025	4.96	0.0070
XMS-CASPT2	4.89	0.0000	4.76	0.0001	4.53	0.0080	4.86	0.0025	4.59	0.0531
EOM-CCSD	5.18	0.0000	5.12	0.0011	4.78	0.0384	5.24	0.0097	5.07	0.0234
ADC(2)	5.25	0.0000	5.16	0.0013	4.71	0.0464	5.26	0.0152	5.04	0.0323
ADC(3)	4.98	0.0000	4.91	0.0013	4.59	0.0389	5.05	0.0092	4.89	0.0227
B3LYP	5.50	0.0000	5.31	0.0017	4.80	0.0501	5.43	0.0133	5.20	0.0330
CAM-B3LYP	5.66	0.0000	5.43	0.0021	5.04	0.0561	5.60	0.0147	5.39	0.0359
M06-2X	5.71	0.0000	5.51	0.0021	5.10	0.0573	5.67	0.0153	5.47	0.0361
ωB97X	5.69	0.0000	5.49	0.0022	5.12	0.0576	5.63	0.0155	5.44	0.0369
Diabatic										
MRCI	5.05	0.0029	5.47	0.0066	4.84	0.0033	5.45	0.0042	5.01	0.0080
XMS-CASPT2	4.91	0.0048	5.21	0.0097	4.80	0.0044	5.38	0.0074	4.99	0.0118
Expt.	4.88	0.0006	4.62	0.0050	3.69	0.0355	4.73	0.0076	4.56	0.0161

a Experimental data taken from ref (62).

**Figure 7:**
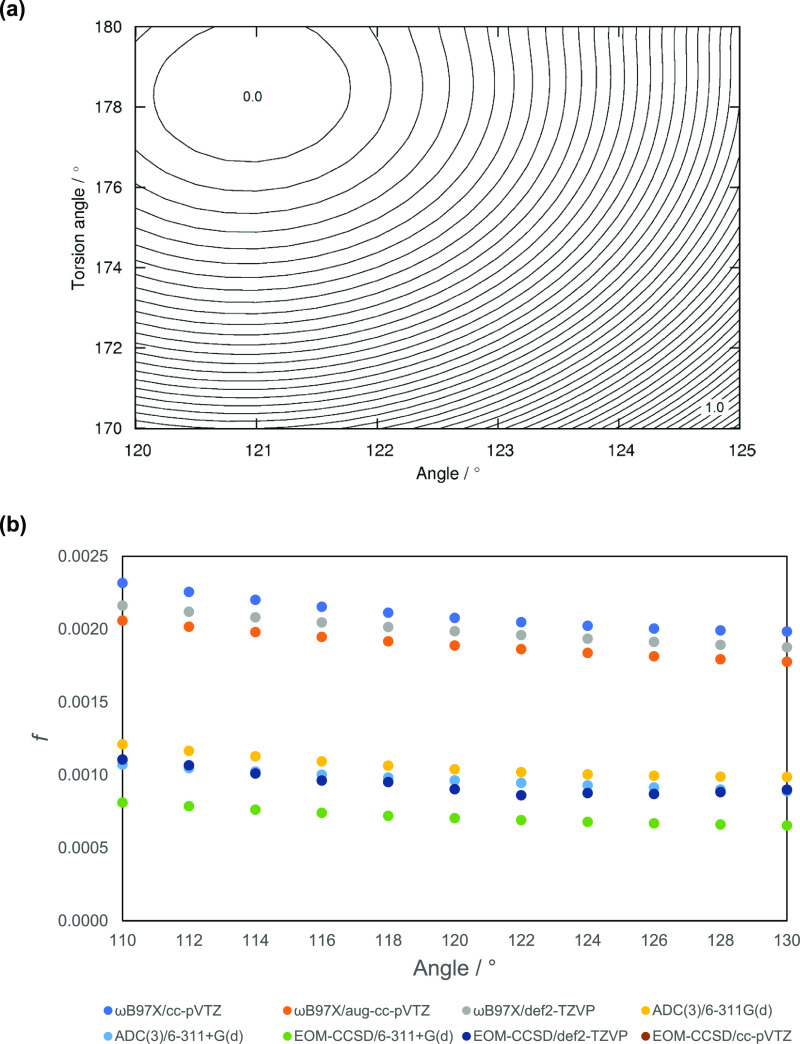
(a) Two-dimensional potential energy surface scanned along the torsion angle C(aromatic)–C(aromatic)–C(aromatic)–C(methyl) and the bond angle C(aromatic)–C(aromatic)–C(methyl); kcal mol^–1^, contour value of 0.025 kcal mol^–1^. (b) Calculated oscillator strength as a function of the bond angle C(aromatic)–C(aromatic)–C(methyl) (see key for details of the methods).

For each of the molecules considered, the point-group symmetry of the geometry of the S_0_ state is *D*_6*h*_ (benzene), *C_s_* (toluene), *C*_2*v*_ (aniline), *C*_2*v*_ (fluorobenzene), and *C_s_* (phenol). Breaking of the planar aromatic ring would therefore be assumed to be responsible for an enhancement in the oscillator strength of the S_1_ ← S_0_ transition. The effect of symmetry breaking upon the calculated oscillator strength is given in Figure 7 for toluene. As the torsion angle (between three aromatic carbon atoms and the methyl carbon) is decreased by ∼10°, the energy of the S_0_ state increases by only 1 kcal mol^–1^ (Figure 7a). As such, there is effectively little to no barrier to symmetry breaking at finite temperature. While there is a small change in the oscillator strength as the symmetry of the molecule is broken, this is a small effect (Figure 7b).

We now consider the extent to which the S_1_ and S_2_ states are mixed in the pseudo-diabatization procedure. In Table 4 are the calculated diabatic rotation angles for MRCI and XMS-CASPT2 for each of the molecules considered. While these rotation angles have an effect on the diabatic energies (eq 7), the effect on the oscillator strengths is determined by the mixing of the CI coefficients. As noted above, the coupling between the S_2_ and S_1_ states is strong for aniline with analytic NACMEs at the S_0_ geometry, in contradiction to the rotation angle calculated using the approximate pseudo-diabatization procedure. This provides further evidence that, in the event of strong coupling, the pseudo-diabatization procedure becomes less reliable.

**Table 4:** Diabatic Rotation Angles Determined Using the Pseudo-diabatization Procedure^a^

molecule	θ (MRCI)	θ (XMS-CASPT2)
benzene	0.02	–0.01
toluene	–24.6	–20.6
aniline	0.1	0.1
fluorobenzene	–20.8	–21.8
phenol	8.7	11.1

a All angles in °.

## Conclusions

We have applied a simple pseudo-diabatization scheme to benzene, toluene, and three other monosubstituted benzenes to account for the vibronic coupling between the S_2_ and S_1_ states and the effect this has upon the transition properties of the S_1_ ← S_0_ excitation using multireference approaches. In the adiabatic basis, MRCI and XMS-CASPT2 exhibit oscillator strengths lower than the experimental value. Inclusion of approximate vibronic coupling effects through the pseudo-diabatic states results in improved quantitative values of the oscillator strength for all molecules except aniline. In this case, the vibronic coupling was determined to be strong relative to that seen in the other molecules; the success of the simple approach adopted here is predicated on weak coupling of the S_2_ and S_1_ states; in the case of aniline, this coupling is strong, leading to a poor description of the oscillator strength.

## Supporting Information Available

Potential energy scans for aniline, NACME magnitudes, and harmonic vibrational frequency scaling data (PDF)
