# Supplementary material for: Benzene, Toluene, and Monosubstituted Derivatives: Diabatic Nature of the Oscillator Strengths of S1 ← S0 Transitions
Source: J Phys Chem A. 2021 Jun 16;125(24):5237–45. doi: 10.1021/acs.jpca.1c01685 (PMC8279645; doi:10.1021/acs.jpca.1c01685)
Supplement: Supplementary file 1 — jp1c01685_si_001.pdf [file jp1c01685_si_001.pdf]

Benzene, Toluene and Mono-Substituted Derivatives: Diabatic Nature of the Oscillator Strengths of $S_1 \leftarrow S_0$ Transitions

David Robinson[(a)]*, Saleh S. Alarfaji[(b)] and Jonathan D. Hirst[(b)]

(a) Department of Chemistry and Forensics, School of Science and Technology, Nottingham Trent University, Clifton Lane, Nottingham, NG11 8NS, United Kingdom.

(b) School of Chemistry, University of Nottingham, University Park, Nottingham, NG7 2RD, United Kingdom.

* david.robinson@ntu.ac.uk

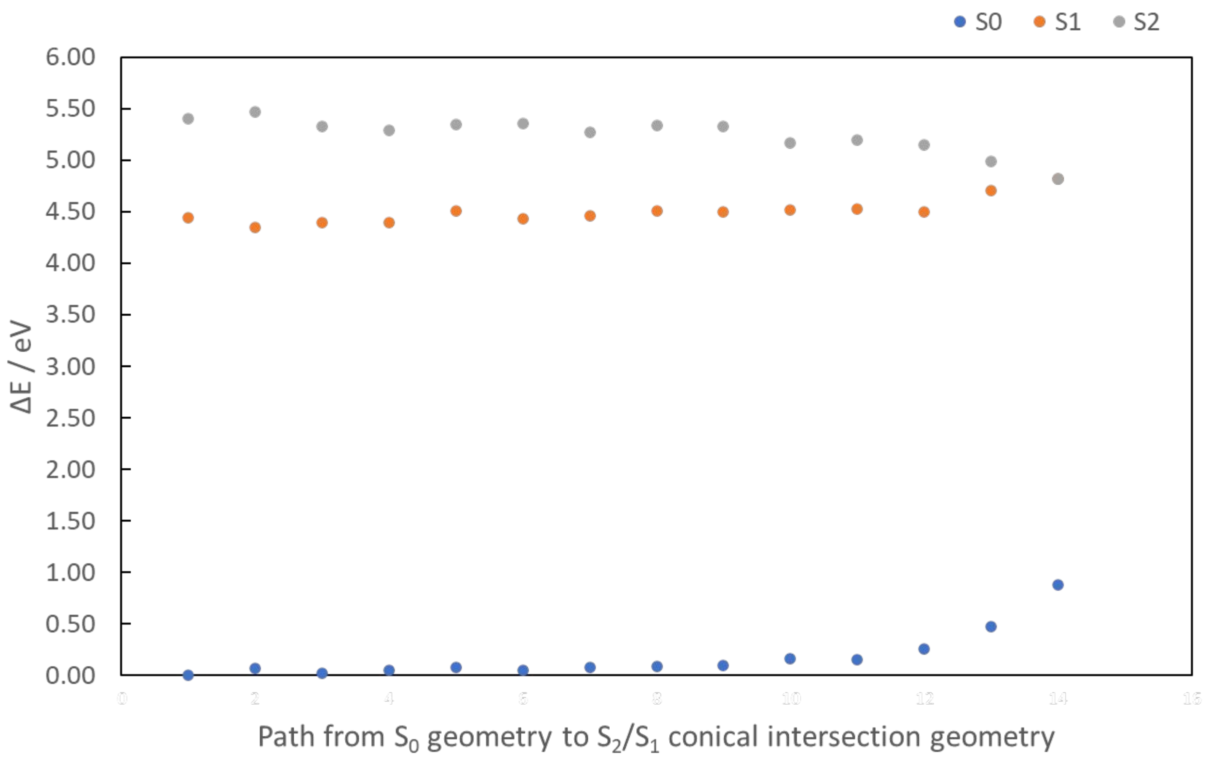

Figure S1. Potential energy scans of aniline along the S_0 minimum to S_2/S_1 conical intersection deformation coordinate for the S_0 state (blue), S_1 (orange) and S_2 (grey).

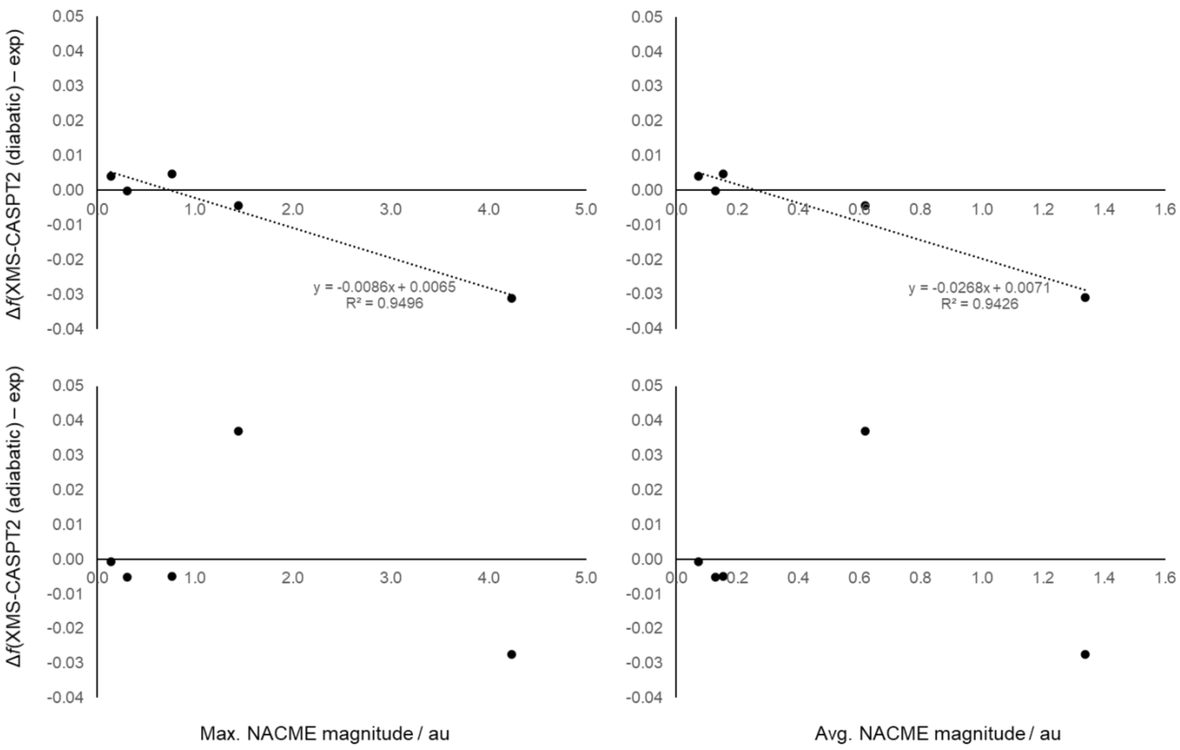

Figure S2. Maximum and average NAMCE magnitudes versus the difference in oscillator strength (calculated and experimental).

Harmonic vibrational frequency scaling

Experimental vibrational frequencies for a series of diatomic, triatomic and polyatomic molecules[1-14] have been used as a benchmark (see Table S1). Tables S2(a)-S2(b) give the calculated harmonic frequencies for the diatomic molecules, along with the experimentally determined values. The convergence of the vibrational frequencies is slow with respect to the increasing number of basis functions. This is seen most obviously for the case of OH^- (Figure S1). The addition of both higher angular momentum terms and diffuse functions are required to achieve a converged result; this is 112 cm^{-1} higher than the experimental value for OH^-, suggesting significant anharmonicity.

Table S2(e) presents the differences between the calculated harmonic and anharmonic frequencies at the extrapolated CBS limit. The molecules which have a bond to a hydrogen atom have the largest difference between the harmonic and anharmonic frequencies. Furthermore, the CASPT2/CBS estimate for the anharmonic frequency for H_2 is in much worse agreement with the experimental value than that of the harmonic frequency. The CASPT2/CBS differences from experiment are shown in Figure S2. For the majority of molecules, the calculated harmonic vibrational frequencies underestimate

the experimental values, with the exception of OH, OH^-, HF and NO^-. The calculated anharmonic frequencies are in much better agreement with experiment than the harmonic frequencies (see Table S2(c)-S2(d)).

For triatomics, Tables S3(a)-(c) give the calculated harmonic frequencies for H_2O, SO_2, CO_2, HCN, HNC and O_3, along with the experimentally determined values. The results and observations display large similarities to those above for the diatomic molecules; much larger deviations from experiment are seen when bonds to hydrogen atoms are involved in the vibrational mode, and relatively slow convergence of the vibrational frequencies with respect to increasing number of basis functions. This is typified by the examples of O_3 and H_2O. For O_3, the maximum deviation from experiment of the vibrational frequencies at the CBS limit is <25 cm^{-1}, while for H_2O, the smallest deviation is ~88 cm^{-1}. For both molecules, much larger deviations are seen with smaller basis sets. Final scaling parameters are given in Table S5.

Molecule	Active space	Molecule	Active space
H_2	(2,2)	SO_2	(18,12); (4,4)
C_2	(8,8)	CO_2	(16,12); (8,6)
N_2	(10,8)	HCN	(10,9); (4,4)
O_2	(12,8)	HNC	(10,9)
F_2	(14,8)	O_3	(18,12)
Cl_2	(14,8)	Ethene	(12,12)[(a)]; (2,2)
OH	(7,5)	CH_3	(7,7)
OH^-	(8,5)	Methane	(8,8)
HF	(8,5)	Formaldehyde	(12,10)
HCl	(8,5)	Furan	(6,5)
NO	(11,8)	NH_3	(8,7)
NO^-	(12,8)	Trans-butadiene	(4,4)
CO	(10,8)	Acetaldehyde	(2,2)
ClF	(14,8)	Cyclopentadiene	(4,4)
H_2O	(8,6)	1,3-pentadiene	(4,4)

Table S1. Molecules studied and their respective active space in the CASSCF/CASPT2 calculations. [(a)]Two active spaces were used for ethane; one including all valence electrons and orbitals, and the second containing just the π electrons and orbitals.

Molecule	6-31G(d)	6-31G(d,p)	6-31+G(d)	6-31+G(d,p)	6-31++G(d,p)	6-311G(d)	6-311G(d,p)	6-311+G(d)	6-311+G(d,p)
H_2	4333.6	4450.0	4333.6	4450.0	4436.9	4247.6	4372.8	4247.6	4372.8
C_2	1868.3	1868.3	1852.4	1852.4	1852.4	1828.0	1828.0	1819.3	1819.3
N_2	2324.6	2324.6	2319.9	2319.9	2319.9	2324.4	2324.4	2319.4	2319.4
O_2	1549.6	1549.6	1529.9	1529.9	1529.9	1578.6	1578.6	1562.3	1562.3
F_2	861.5	861.5	832.0	832.0	832.0	754.7	754.7	743.7	743.7
Cl_2	487.8	487.8	484.2	484.2	484.2	490.6	490.6	497.7	497.7
OH	3601.7	3736.4	3585.5	3731.5	3729.2	3660.6	3747.8	3652.2	3739.3
OH^-	3400.3	3557.8	3626.2	3795.6	3772.1	3430.9	3591.3	3647.0	3812.4
HF	3971.4	4157.5	3902.1	4116.1	4115.4	4051.4	4231.7	4020.4	4199.3
HCl	2951.5	3041.7	2938.0	3034.6	3034.4	2852.9	3004.8	2856.3	3003.5
NO	1869.8	1869.8	1854.6	1854.6	1854.6	1896.7	1896.7	1883.5	1883.5
NO^-	1410.0	1410.0	1349.7	1349.7	1349.7	1417.5	1417.5	1368.0	1368.0
CO	2141.6	2141.6	2132.9	2132.9	2132.9	2156.3	2156.3	2145.6	2145.6

ClF	745.9	745.9	742.0	742.0	742.0	673.7	673.7	686.2	686.2

Table S2(a). CASPT2 harmonic frequencies (cm^{-1}) calculated with Pople-type basis sets.

Molecule	cc-pVDZ	cc-pVTZ	cc-pVQZ	aug-cc-pVDZ	aug-cc-pVTZ	aug-cc-pVQZ	aug-cc-pV5Z	CBS[a]	Expt.[b]
H_2	4337.7	4378.8	4376.8	4304.7	4371.4	4372.4	4376.5	4376.4	4401.2
C_2	1828.6	1835.7	1844.0	1808.1	1827.2	1839.9	1843.6	1848.5	1855.1
N_2	2323.0	2324.6	2332.4	2298.8	2316.3	2329.9	2334.6	2339.7	2358.6
O_2	1564.2	1555.1	1565.9	1537.1	1543.3	1562.2	1565.2	1572.7	1580.2
F_2	772.3	892.0	888.3	801.2	885.7	887.9	893.0	893.3	916.9
Cl_2	496.7	534.2	541.7	489.3	530.4	540.7	548.0	551.4	559.7
OH	3682.0	3730.6	3742.6	3678.2	3713.5	3736.3	3740.3	3749.3	3737.8
OH^-	3423.4	3685.5	3765.9	3763.8	3814.0	3836.0	3842.4	3850.8	3738.4
HF	4139.8	4196.6	4185.2	4095.9	4157.9	4170.4	4171.3	4176.4	4138.4
HCl	2992.6	2979.1	2979.6	2949.3	2973.5	2975.5	2981.7	2981.7	2990.9
NO	1891.2	1874.0	1880.5	1849.0	1859.0	1875.2	1878.4	1884.7	1904.2
NO^-	1417.6	1396.0	1394.5	1330.1	1348.3	1362.2	1364.2	1369.8	1363.0
CO	2135.7	2141.4	2150.8	2096.6	2131.9	2146.5	2149.8	2155.5	2169.8

ClF	692.1	758.7	770.5	741.0	762.2	772.0	776.6	780.2	783.5

Table S2(b). CASPT2 harmonic frequencies (cm^{-1}) calculated with the Dunning correlation-consistent basis sets.

Molecule	6-31G*	6-31G**	6-31+G*	6-31+G**	6-31++G**	6-311G*	6-311G**	6-311+G*	6-311+G**
H_2	4123.5	4252.0	4123.5	4252.0	4237.1	4053.8	4185.4	4053.8	4185.4
C_2	1855.2	*1855.2*	1843.2	*1843.2*	*1843.2*	1830.0	*1830.0*	1815.5	*1815.5*
N_2	2318.2	*2318.2*	2313.4	*2313.4*	*2313.4*	2318.9	*2318.9*	2313.8	*2313.8*
O_2	1561.3	*1561.3*	1541.9	*1541.9*	*1541.9*	1589.6	*1589.6*	1573.7	*1573.7*
F_2	891.6	*891.6*	863.1	*863.1*	*863.1*	791.6	*791.6*	780.9	*780.9*
Cl_2	531.4	*531.4*	528.0	*528.0*	*528.0*	534.0	*534.0*	540.9	*540.9*
OH	3473.8	3605.2	3462.4	3603.8	3602.1	3511.5	3617.3	3506.3	3612.7
OH^-	3250.5	3407.0	3484.5	3652.0	3627.4	3265.5	3434.8	3489.4	3669.0
HF	3842.0	3989.6	3800.3	3961.4	3961.6	3859.1	4054.7	3840.0	4031.2
HCl	2869.7	2967.8	2857.4	2961.0	2960.8	2787.5	2936.7	2790.2	2935.4
NO	1871.5	*1871.5*	1856.6	*1856.6*	*1856.6*	1897.6	*1897.6*	1884.6	*1884.6*
NO^-	1425.4	*1425.4*	4149.7	*4149.7*	*4149.7*	1434.9	*1434.9*	1393.7	*1393.7*
CO	2140.3	*2140.3*	2131.5	*2131.5*	*2131.5*	2154.9	*2154.9*	2144.3	*2144.3*
ClF	781.9	*781.9*	778.2	*778.2*	*778.2*	712.4	*712.4*	724.5	*724.5*

Table S2(c). Calculated anharmonic frequencies (cm^{-1}) for the diatomic molecules using the Pople-type basis sets.

Molecule	cc-pVDZ	cc-pVTZ	cc-pVQZ	aug-cc-pVDZ	aug-cc-pVTZ	aug-cc-pVQZ	aug-cc-pv5z	CBS	Experimental
H_2	4154.2	4190.1	4190.0	4123.2	4180.7	4185.3	4189.4	4190.8	4401.2
C_2	1827.9	1823.1	1844.4	1800.1	1862.9	1870.9	1867.9	1871.5	1855.1
N_2	2316.8	2319.0	2326.7	2293.1	2310.8	2324.3	2328.8	2333.9	2358.6
O_2	1574.8	1567.7	1577.9	1548.6	1556.0	1574.2	1577.1	1584.4	1580.2
F_2	806.7	921.4	918.3	834.2	915.4	917.9	922.8	923.2	916.9
Cl_2	540.0	576.5	583.8	532.9	572.8	582.9	589.9	593.3	559.7
OH	3555.2	3608.3	3620.0	3552.4	3595.0	3614.5	3618.4	3626.0	3737.8
OH^-	3275.6	3537.8	3620.8	3625.7	3681.3	3700.7	3707.1	3714.4	3738.4
HF	3976.6	4040.8	4029.1	3934.5	4010.3	4016.7	4017.4	4019.9	4138.4
HCl	2924.4	2912.5	2914.4	2884.6	2906.9	2910.7	2915.8	2916.7	2990.9
NO	1892.1	1876.2	1882.7	1851.0	1861.5	1877.5	1880.7	1887.0	1904.2
NO^-	1432.5	1415.7	1415.5	1347.1	1370.1	1383.7	1385.9	1391.3	1363.0
CO	2135.0	2140.5	2149.5	2096.2	2130.9	2145.2	2148.4	2154.0	2169.8
ClF	729.7	794.5	806.0	777.1	797.9	807.5	812.0	815.4	783.5

Table S2(d). Calculated anharmonic frequencies (cm^{-1}) for the diatomic molecules using the Dunning correlation-consistent basis sets.

Molecule	$\omega_{harmonic} - \omega_{anharmonic}$ / cm^{-1}
H_2	185.6
C_2	-23.1
N_2	5.8
O_2	-11.7
F_2	-29.9
Cl_2	-41.9
OH	123.3
OH^-	136.4
HF	156.5
HCl	65.0
NO	-2.3
NO^-	-21.5
CO	1.6
ClF	-35.3

Table S2(e). Difference between the calculated harmonic and anharmonic frequencies for CASPT2 / CBS.

Molecule	6-31G*	6-31G**	6-31+G*	6-31+G**	6-31++G**	6-311G*	6-311G**	6-311+G*	6-311+G**
H_2O	3703.4	3846.4	3691.1	3837.9	3833.7	3798.9	3861.1	3782.9	3852.4
	1747.2	1699.1	1709.4	1653.8	1650.4	1766.7	1700.6	1736.7	1674.3
	3801.3	3941.4	3785.7	3931.8	3928.0	3848.6	3894.4	3829.2	3889.2
SO_2	1088.6	*1088.6*	1076.7	*1076.7*	*1076.7*	1100.5	*1100.5*	1089.8	*1089.8*
	492.5	*492.5*	487.7	*487.7*	*487.7*	501.1	*501.1*	495.9	*495.9*
	1272.6	*1272.6*	1236.7	*1236.7*	*1236.7*	1284.8	*1284.8*	1258.2	*1258.2*
CO_2	1334.4	*1334.4*	1322.1	*1322.1*	*1322.1*	1343.9	*1343.9*	1338.3	*1338.3*
	2395.5	*2395.5*	2364.2	*2364.2*	*2364.2*	2404.5	*2404.5*	2381.9	*2381.9*
	636.7	*636.7*	632.3	*632.3*	*632.3*	654.9	*654.9*	652.9	*652.9*
HCN	3438.0	3469.9	3511.9	3537.2	3536.7	3404.9	3426.9	3488.5	3502.4
	2108.0	2108.5	2101.0	2100.5	2100.4	2091.2	2092.0	2087.1	2087.0
	698.7	703.0	665.7	673.1	670.8	713.6	737.0	678.8	707.4
HNC	3795.2	3847.3	3768.4	3823.5	3823.0	3796.4	3803.8	3778.2	3794.0
	2050.9	2049.9	2041.0	2039.6	2039.5	2035.9	2034.6	2026.5	2025.8
	480.0	464.2	473.0	463.1	464.7	463.0	489.8	434.8	486.2
O_3	1057.6	*1057.6*	1049.7	*1049.7*	*1049.7*	1090.1	*1090.1*	1082.8	*1082.8*
	662.0	*662.0*	658.8	*658.8*	*658.8*	697.0	*697.0*	694.3	*694.3*
	967.5	*967.5*	953.2	*953.2*	*953.2*	1000.6	*1000.6*	988.3	*988.3*

Table S3(a). Calculated harmonic vibrational frequencies (cm^{-1}) for triatomic molecules using the Pople basis sets.

Molecule	cc-pVDZ	cc-pVTZ	cc-pVQZ	aug-cc-pVDZ	aug-cc-pVTZ	aug-cc-pVQZ	CBS	Experimental	Mode symmetry
H_2O	3804.8	3832.8	3839.4	3782.3	3810.3	3828.9	3830.5	3657.0	A_1
	1699.9	1693.8	1689.4	1658.4	1676.6	1682.0	1685.0	1595.0	A_1
	3874.2	3856.2	3864.3	3853.5	3833.8	3854.3	3843.7	3756.0	B_2
SO_2	1050.4	1129.6	1167.0	1026.8	1117.3	1155.8	1167.2	1151.4	A_1
	486.0	505.6	509.5	467.9	499.0	508.0	513.1	517.7	A_1
	1224.0	1302.0	1579.4	1164.4	1222.8	1445.7	1397.1	1361.8	B_2
CO_2	1331.0	1335.2	1339.8	1308.2	1330.0	1337.4	1340.7	1333.0	Σ_g
	2388.2	2378.2	2376.2	2328.6	2354.9	2368.4	2371.1	2349.0	Σ_g
	647.2	654.6	663.5	656.1	658.3	662.8	662.1	667.0	Π_u
HCN	3419.0	3418.8	3413.2	3474.6	3490.4	3489.5	3493.7	3312.0	Σ
	2085.8	2093.7	2104.1	2061.8	2088.8	2100.7	2103.9	2089.0	Σ
	701.8	705.4	710.9	681.9	699.5	703.2	706.5	712.0	Π
HNC	3769.9	3799.6	3798.9	3754.7	3783.7	3793.4	3797.8	3652.9	Σ
	2025.4	2035.7	2043.5	2001.0	2027.6	2039.0	2042.3	2029.0	Σ
	464.1	483.1	476.2	421.8	464.7	467.9	477.6	477.0	Π
O_3	1061.3	1087.5	1101.1	1054.7	1080.9	1099.7	1100.8	1110.0	A_1
	681.9	689.5	697.1	678.6	686.9	696.9	696.1	705.0	A_1
	953.2	1000.1	1018.0	941.3	986.9	1014.5	1017.9	1042.1	B_2

Table S3(b). Calculated harmonic vibrational frequencies (cm^{-1}) for triatomic molecules using the Dunning correlation-consistent basis sets.

Molecule	Mode symmetry	Full-valence		Reduced active space		Experiment
	A_1	1167.2	*(15.8)*	1149.5	(-1.9)	1151.4
SO_2	A_1	513.1	*(-4.6)*	519.2	(1.5)	517.7
	B_2	1397.1	*(35.3)*	1356.4	(-5.4)	1361.8
	Σ_g	1340.7	*(7.7)*	1345.7	*(12.7)*	1333.0
CO_2	Σ_g	2371.1	*(22.1)*	2388.6	*(39.6)*	2349.0
	Π_u	662.1	*(-4.9)*	620.7	*(-46.4)*	667.0
	Σ	3493.7	*(181.7)*	3488.9	*(176.9)*	3312.0
HCN	Σ	2103.9	*(14.9)*	2121.7	*(32.7)*	2089.0
	Π	706.5	*(-5.5)*	672.6	*(-39.4)*	712.0

Table S3(c). Calculated harmonic frequencies (cm^{-1}) using a full valence active space and a reduced active space (see main text for details) with CASPT2 / CBS. Differences (cm^{-1}) from experiment are given in parentheses.

Molecule	6-31G*	6-31G**	6-31+G*	6-31+G**	6-31++G**	6-311G*	6-311G**	6-311+G*	6-311+G**
CH_3	3301.3	3158.4	3117.4	3150.2	3150.1	3081.2	3093.3	3078.6	3091.6
	442.9	426.6	500.7	489.2	488.1	456.3	443.3	483.6	474.6
	3301.4	3344.2	3293.2	3335.8	3335.7	3261.8	3275.5	3259.1	3273.8
	1440.4	1449.7	1431.4	1440.7	1438.4	1414.5	1413.1	1412.3	1411.8
CH_4	3031.1	3066.3	3021.9	3056.6	3056.0	3003.1	3012.4	3000.5	3010.9
	1595.4	1598.9	1575.3	1576.9	1567.2	1577.7	1558.3	1575.2	1554.4
	3154.9	3201.0	3143.1	3189.7	3188.9	3127.1	3138.6	3124.3	3136.5
	1386.8	1381.9	1378.6	1373.7	1373.4	1363.2	1344.8	1361.8	1344.0
Formaldehyde	2927.0	2948.8	2918.3	2946.8	3054.8	2901.2	2886.9	2871.7	2880.9
	1777.7	1796.9	1772.0	1772.2	1772.3	1786.6	1784.4	1776.8	1773.7
	1552.8	1560.1	1538.2	1544.9	1570.7	1538.9	1539.5	1527.1	1531.5
	1176.1	1177.9	1164.4	1174.1	1188.8	1160.4	1171.1	1157.2	1168.1
	2991.5	3582.4	3405.5	3259.7	3137.6	4200.3	3647.8	3405.1	3264.7
	1275.9	1310.7	1291.3	1285.3	1284.1	1311.5	1302.4	1289.8	1280.6
Furan	3351.5	3383.6	3346.4	3379.8	3379.5	3326.8	3330.3	3323.0	3328.2
	3322.2	3355.6	3313.6	3349.4	3348.9	3299.5	3303.0	3294.7	3300.8
	1541.5	1545.4	1525.5	1529.0	1528.8	1521.5	1516.0	1514.5	1510.1
	1451.6	1455.8	1441.6	1445.1	1444.8	1425.4	1420.3	1420.5	1417.0
	1185.8	1184.6	1178.1	1176.9	1176.7	1171.3	1165.9	1168.8	1163.9
	1122.6	1122.6	1112.5	1112.4	1112.4	1118.2	1116.8	1110.7	1109.8
	1042.8	1042.7	1034.1	1033.9	1033.9	1031.5	1026.6	1027.3	1023.5
	878.8	878.2	874.6	874.0	873.9	881.5	878.3	880.5	877.6
	762.3	782.4	737.4	758.4	726.8	755.5	785.0	736.7	769.9
	648.9	658.0	633.0	644.4	620.4	652.0	667.8	633.6	652.7
	550.7	553.2	527.6	532.4	446.5	553.1	555.2	525.2	530.5
	766.9	783.1	753.0	769.9	752.7	766.8	792.2	753.0	781.2
	709.1	715.2	695.9	703.7	696.0	710.8	718.6	698.5	709.5

	607.9	608.3	592.0	592.1	589.1	610.1	609.2	594.4	593.1
	3346.7	3377.8	3342.0	3374.7	3374.3	3321.1	3324.0	3317.7	3322.1
	3311.5	3344.9	3302.5	3338.3	3337.4	3288.4	3292.0	3283.7	3289.7
	1617.9	1621.6	1601.8	1604.7	1604.5	1588.1	1583.9	1581.2	1577.5
	1315.0	1318.9	1307.7	1310.9	1310.1	1293.7	1287.8	1293.7	1289.8
	1248.1	1248.5	1235.4	1236.3	1236.3	1237.0	1237.0	1231.1	1231.5
	1088.1	1088.3	1079.2	1079.6	1079.3	1068.9	1064.5	1067.4	1063.5
	887.2	885.8	885.4	884.3	883.9	885.4	882.2	885.6	882.7
NH3	3395.9	3469.1	3394.9	3464.8	3464.2	3437.6	3438.8	3428.7	3435.9
	1238.8	1203.9	1186.1	1134.4	1143.6	1253.9	1228.1	1197.6	1177.6
	3477.8	3551.1	3471.6	3540.4	3539.9	3477.5	3475.9	3463.8	3464.8
	1734.1	1703.2	1721.1	1683.0	1682.3	1743.7	1651.0	1730.5	1636.4
Trans-butadiene	3314.6	3349.4	3306.1	3342.4	3341.8	3288.1	3288.6	3284.3	3287.1
	3221.7	3250.8	3212.5	3243.3	3243.0	3193.9	3193.9	3190.1	3191.8
	3208.9	3239.6	3200.0	3233.0	3232.7	3183.8	3183.4	3180.2	3181.7
	1714.0	1717.8	1700.2	1703.6	1703.2	1687.7	1679.9	1681.9	1674.7
	1522.0	1521.8	1515.9	1514.4	1513.8	1497.7	1486.2	1495.8	1486.8
	1338.4	1336.5	1331.7	1329.4	1328.4	1318.8	1309.7	1317.0	1309.0
	1263.1	1258.8	1259.5	1254.4	1253.6	1245.7	1234.0	1244.9	1234.6
	923.4	922.6	921.1	919.6	919.0	912.0	906.5	911.0	906.3
	523.7	523.5	520.9	518.1	517.6	519.5	517.6	519.2	514.3
	1023.1	1022.6	1005.9	1012.7	1002.8	1009.8	1002.3	1000.8	1003.3
	862.4	860.3	847.4	842.3	790.9	844.2	845.2	829.9	830.8
	520.6	518.4	514.3	514.7	501.6	516.6	514.3	511.3	511.9
	160.4	159.3	141.0	140.4	129.2	158.0	157.0	142.9	141.0
	965.3	968.2	949.6	952.6	941.2	952.0	954.4	945.8	948.0
	863.3	861.1	847.6	842.5	791.4	844.3	845.4	831.6	832.4
	737.0	730.7	723.4	715.9	677.7	732.3	723.4	723.7	712.9
	3314.8	3349.7	3306.3	3342.6	3342.0	3288.3	3288.9	3284.5	3287.4

	3222.2	3252.8	3213.3	3246.3	3246.0	3198.6	3198.8	3195.1	3197.2
	3215.7	3244.7	3207.4	3237.8	3237.5	3187.1	3186.3	3183.4	3184.4
	1663.6	1667.3	1649.5	1652.6	1652.2	1636.9	1629.1	1630.5	1623.8
	1448.1	1446.7	1440.8	1438.6	1438.1	1424.7	1416.9	1421.5	1415.6
	1350.2	1344.1	1346.6	1339.5	1338.2	1334.0	1315.9	1333.0	1317.9
	1027.5	1029.3	1026.3	1020.3	1019.3	1017.0	1016.1	1016.3	1007.9
	298.1	296.1	299.0	296.4	295.3	298.2	294.1	298.3	294.7
Acetaldehyde	3237.7	3270.1	3224.6	3258.6	3257.9	3209.6	3207.9	3206.5	3206.1
	3107.1	3133.1	3095.3	3121.6	3120.8	3080.7	3078.8	3077.2	3076.7
	3019.8	3037.3	3025.2	3045.1	3043.5	2981.0	2970.6	2987.8	2978.0
	1807.7	1808.8	1783.1	1783.5	1783.3	1799.5	1797.6	1784.8	1782.8
	1529.1	1522.5	1514.4	1509.3	1507.5	1505.7	1485.2	1503.0	1485.1
	1470.4	1469.2	1459.7	1456.5	1456.6	1456.3	1451.7	1451.8	1445.7
	1441.2	1432.7	1436.7	1426.7	1426.6	1417.3	1395.4	1418.8	1396.8
	1169.4	1165.4	1167.4	1163.8	1162.7	1156.8	1146.4	1157.8	1147.9
	930.0	926.1	929.0	924.1	923.8	913.9	908.7	916.1	909.9
	515.9	512.6	517.7	514.2	513.8	515.3	511.0	516.6	512.3
	3187.8	3220.4	3174.8	3208.9	3207.8	3162.2	3162.3	3157.8	3159.1
	1535.9	1532.1	1517.3	1514.3	1510.8	1510.2	1494.7	1506.7	1491.6
	1156.3	1150.1	1148.4	1143.1	1141.5	1141.9	1131.0	1139.1	1128.2
	795.9	792.6	793.0	789.5	787.7	786.5	783.6	785.9	782.0
	148.7	150.5	137.9	138.6	139.3	151.4	155.4	142.0	144.8
Cyclopentadiene	3292.2	3326.0	3283.6	3319.7	3318.9	3267.5	3271.8	3263.5	3269.8
	3270.3	3303.4	3261.6	3297.2	3296.1	3244.8	3248.3	3240.8	3246.6
	3092.6	3121.1	3081.9	3110.8	3110.3	3071.9	3071.3	3068.2	3069.4
	1569.9	1571.6	1554.1	1555.4	1555.1	1538.5	1534.3	1531.0	1527.2
	1489.0	1480.1	1473.2	1465.1	1462.4	1456.5	1439.1	1451.3	1436.0
	1442.2	1444.5	1434.0	1435.3	1434.6	1416.7	1409.7	1414.2	1408.8
	1157.4	1154.7	1151.3	1147.8	1147.6	1144.1	1129.2	1135.7	1128.3

	1048.3	1047.7	1044.5	1043.4	1043.2	1033.0	1030.2	1032.3	1029.7
	961.5	960.8	957.0	955.7	955.5	948.8	945.3	947.9	944.5
	814.7	812.5	813.1	810.8	810.8	807.4	803.1	807.4	803.2
	1142.4	1139.4	1128.5	1122.5	1119.3	1121.1	1112.5	1119.9	1112.2
	853.2	867.9	834.0	851.6	837.5	846.9	870.3	841.6	868.0
	662.9	663.6	651.9	653.9	644.8	654.2	654.0	649.9	650.9
	485.5	484.7	475.0	476.7	470.7	486.4	486.6	478.8	480.4
	3137.2	3169.6	3125.4	3159.7	3159.3	3117.3	3117.2	3112.9	3115.0
	938.9	932.5	928.4	923.1	922.0	913.4	911.3	907.9	907.5
	881.9	891.8	874.0	885.5	876.4	875.7	889.2	876.4	890.7
	664.7	667.4	652.0	655.9	646.5	656.3	659.8	651.9	656.0
	330.9	327.2	322.4	320.3	319.9	321.4	318.2	316.9	315.2
	3286.6	3319.7	3277.8	3313.2	3312.1	3260.9	3265.2	3256.7	3263.1
	3261.6	3294.7	3252.9	3288.7	3286.9	3236.4	3239.6	3232.6	3238.1
	1649.2	1651.5	1632.6	1634.5	1634.2	1619.4	1615.2	1610.9	1607.5
	1354.1	1356.8	1347.1	1349.1	1348.6	1331.4	1325.9	1329.6	1325.7
	1314.3	1310.5	1309.1	1304.6	1304.5	1290.4	1279.6	1289.1	1279.1
	1152.7	1147.3	1136.4	1132.7	1132.5	1137.5	1125.5	1134.3	1118.0
	1009.0	1005.8	1004.4	1000.6	1000.4	995.5	990.3	994.1	989.1
	819.4	817.8	818.4	816.8	816.6	813.5	809.1	814.2	810.0
	3313.2	3348.4	3304.3	3340.9	3340.2	3286.7	3287.5	3282.5	3285.8
	3220.2	3249.7	3210.9	3242.4	3242.0	3194.0	3194.3	3190.3	3192.4
	3211.1	3241.7	3202.6	3235.0	3234.7	3185.9	3185.4	3181.9	3183.5
	3205.9	3237.5	3196.1	3229.8	3229.4	3182.6	3182.5	3178.9	3180.7
1,3-pentadiene	3196.9	3229.0	3186.4	3221.2	3221.4	3175.0	3175.5	3170.9	3173.4
	3190.3	3222.6	3178.1	3211.8	3210.7	3165.7	3163.8	3162.3	3161.9
	3093.6	3119.4	3083.5	3109.1	3108.1	3068.4	3065.7	3065.6	3064.5
	1729.6	1732.3	1715.6	1717.9	1717.4	1703.7	1696.9	1697.4	1691.2
	1677.5	1680.6	1662.5	1665.1	1664.7	1651.2	1643.9	1644.3	1638.0

	1558.3	1552.2	1544.2	1538.8	1536.7	1533.2	1514.6	1530.6	1513.3
	1497.4	1496.1	1490.2	1488.0	1487.3	1473.4	1462.4	1470.6	1462.1
	1471.9	1463.2	1463.9	1454.2	1453.5	1448.5	1426.5	1446.0	1424.8
	1364.5	1358.1	1359.3	1352.0	1351.0	1345.9	1330.5	1344.6	1331.0
	1346.8	1343.3	1340.2	1335.8	1334.4	1327.7	1315.0	1325.3	1314.6
	1334.9	1328.2	1330.5	1323.3	1322.1	1317.3	1298.1	1316.1	1300.1
	1242.9	1239.9	1238.3	1234.8	1234.1	1224.0	1216.2	1223.0	1215.8
	1137.1	1135.8	1132.3	1130.8	1130.1	1122.1	1116.6	1120.8	1115.9
	1017.6	1018.8	1014.5	1009.9	1009.1	1005.6	1004.6	1004.5	996.4
	953.3	956.2	933.6	935.8	928.6	935.3	939.9	927.0	932.1
	487.8	486.7	486.7	485.3	485.3	485.8	482.6	484.9	481.9
	458.4	455.9	458.4	455.2	454.8	456.8	451.8	456.3	451.8
	198.9	197.4	199.4	197.4	196.7	198.5	196.1	198.6	195.9
	3167.1	3198.4	3154.6	3187.5	3186.2	3140.7	3138.7	3137.4	3136.9
	1543.2	1536.3	1523.3	1516.5	1512.9	1517.5	1496.8	1513.4	1492.9
	1083.5	1076.2	1070.3	1063.1	1058.7	1065.9	1055.5	1060.0	1050.3
	1012.8	1013.6	993.6	1000.0	991.3	997.6	995.7	988.8	995.7
	936.9	933.0	932.4	929.2	925.1	923.7	915.4	922.5	915.0
	853.3	850.9	836.7	831.6	787.6	833.0	834.0	821.1	822.3
	788.8	780.4	774.8	764.9	740.4	784.1	777.3	775.4	767.3
	622.7	620.5	608.9	606.8	592.8	615.3	613.6	607.8	605.6
	127.3	126.4	113.8	112.9	107.0	124.9	124.9	115.3	114.5

Table S4(a). Calculated harmonic frequencies (cm^{-1}) for a range of larger molecules using the Pople-type basis sets.

Molecule	cc-pVDZ	cc-pVTZ	cc-pVQZ	aug-cc-pVDZ	aug-cc-pVTZ	CBS	Experimental	Mode symmetry
CH_3	3096.2	3100.7	3107.0	3080.7	3096.9	3106.3	3004.4	A1'
	418.5	514.6	533.3	521.6	522.3	551.7	606.5	A2''
	3289.4	3281.9	3289.1	3269.3	3276.7	3285.3	3160.8	E'
	1393.5	1413.5	1411.9	1390.8	1407.7	1417.3	1396	E'
CH_4	3016.6	3017.0	3020.8	2997.3	3010.7	3019.8	2917	A1
	1541.4	1567.6	1567.9	1528.7	1570.2	1574.3	1534	E
	3155.4	3141.1	3145.8	3129.5	3134.0	3140.9	3019	T2
	1319.3	1334.5	1336.2	1306.5	1340.4	1339.5	1306	T2
Formaldehyde	2892.1	2881.5	2885.4	2989.1	2988.8	2881.6	2782	A1
	1771.9	1780.3	1779.3	1736.8	1765.9	1781.6	1746	A1
	1520.5	1530.3	1525.4	1528.5	1540.5	1529.2	1500	A1
	1170.6	1176.5	1176.3	1168.7	1169.4	1177.8	1167	B1
	2953.2	3171.4	3091.2	3070.7	3067.6	3167.6	2843	B2
	1256.4	1282.6	1245.7	1254.6	1271.2	1262.5	1249	B2
Furan	3340.1	3332.5	3332.4	3331.2	3325.9	3330.5	3161	A1
	3312.9	3306.9	3306.5	3302.0	3299.6	3305.2	3140	A1
	1524.8	1515.1	1512.2	1499.9	1506.3	1510.6	1491	A1
	1427.0	1420.0	1419.4	1409.2	1416.0	1417.9	1384	A1
	1162.8	1165.8	1165.4	1152.0	1160.8	1166.2	1140	A1
	1114.4	1112.1	1109.5	1091.3	1105.2	1109.7	1066	A1
	1018.5	1023.7	1022.4	1009.2	1020.0	1024.0	995	A1
	873.0	875.6	876.5	863.2	872.4	876.9	871	A1
	804.0	827.1	833.2	782.4	825.9	837.1	838	A2
	683.2	704.7	711.3	660.9	698.0	714.8	728	A2
	586.0	602.9	605.5	578.7	597.1	608.9	603	A2
	820.6	852.1	859.4	789.5	844.0	865.1	863	B1
	723.8	739.1	740.6	712.1	736.5	743.9	745	B1

	614.2	619.2	620.3	601.6	615.6	621.3	613	B1
	3333.4	3325.8	3326.3	3325.0	3318.7	3324.3	3154	B2
	3301.2	3296.3	3296.1	3290.3	3288.4	3294.9	3129	B2
	1591.0	1583.8	1583.4	1567.5	1576.4	1581.7	1556	B2
	1283.2	1295.7	1294.3	1269.4	1288.2	1297.7	1267	B2
	1230.8	1229.2	1227.8	1199.5	1221.7	1227.8	1180	B2
	1058.1	1064.6	1064.9	1047.1	1060.6	1066.5	1040	B2
	876.5	882.5	884.7	870.2	881.8	885.5	873	B2
NH3	3404.6	3430.2	3431.0	3388.2	3413.7	3437.1	3337	A1
	1242.7	1236.8	1215.2	1168.4	1201.3	1219.8	950	A1
	3474.4	3427.3	3431.1	3443.6	3402.7	3418.4	3444	E
	1670.9	1662.3	1654.6	1632.6	1645.4	1654.6	1627	E
Trans-butadiene	3304.8	3290.7	3292.8	3290.5	3284.1	3288.7	3087	Ag
	3205.5	3198.3	3197.1	3194.0	3188.7	3195.7	3003	Ag
	3193.3	3187.3	3188.0	3182.1	3179.6	3186.3	2992	Ag
	1688.4	1680.5	1679.8	1670.5	1674.8	1678.0	1630	Ag
	1476.8	1487.0	1485.1	1474.4	1482.6	1488.1	1438	Ag
	1305.2	1310.9	1311.5	1300.3	1307.6	1312.7	1280	Ag
	1230.1	1231.7	1233.1	1226.0	1229.5	1233.1	1196	Ag
	909.2	908.0	908.9	903.6	905.6	908.4	894	Ag
	525.9	535.3	536.0	529.0	527.6	538.1	512	Ag
	996.4	1001.3	1001.6	991.3	996.7	1002.7	1013	Au
	864.6	897.6	904.5	885.1	894.3	910.7	908	Au
	510.7	511.9	513.8	510.8	510.4	513.5	522	Au
	167.0	170.0	170.4	174.8	164.6	171.0	162	Au
	962.2	975.0	977.7	955.7	960.0	980.1	976	Bg
	866.0	897.8	904.5	885.2	895.6	910.4	912	Bg
	737.2	753.9	757.2	742.4	744.4	760.3	770	Bg
	3305.1	3290.9	3292.9	3290.7	3284.2	3288.9	3101	Bu

	3210.4	3204.2	3204.0	3201.2	3194.3	3202.5	3055	Bu
	3195.9	3189.3	3189.8	3183.3	3181.9	3188.0	2984	Bu
	1634.7	1632.1	1631.5	1618.4	1625.8	1631.1	1596	Bu
	1408.5	1417.4	1415.9	1403.5	1412.8	1418.6	1381	Bu
	1306.4	1314.6	1316.6	1306.8	1313.3	1318.0	1294	Bu
	1023.8	1035.0	1036.1	1013.6	1025.9	1038.5	990	Bu
	291.7	290.8	292.3	293.5	289.9	291.6	301	Bu
Acetaldehyde	3224.1	3208.3	3211.5	3201.7	3203.0	3206.7	3014	A'
	3086.4	3078.8	3079.4	3065.7	3072.4	3077.3	2923	A'
	2970.5	2978.5	2980.0	2984.7	2976.4	2981.5	2716	A'
	1802.8	1790.8	1789.1	1753.3	1776.7	1786.6	1743	A'
	1460.8	1478.4	1478.2	1451.6	1482.9	1482.6	1433	A'
	1435.5	1442.2	1437.8	1418.4	1432.4	1440.7	1395	A'
	1377.2	1387.5	1388.7	1367.1	1392.9	1390.9	1352	A'
	1137.6	1143.8	1145.4	1134.5	1145.5	1146.5	1114	A'
	910.4	907.9	908.0	904.5	908.5	907.3	867	A'
	505.2	508.5	509.9	503.1	508.6	510.3	509	A'
	3179.5	3159.8	3161.7	3151.0	3152.8	3156.4	2964	A"
	1474.4	1490.8	1489.9	1462.2	1494.9	1494.2	1431	A"
	1119.2	1132.8	1134.3	1109.9	1132.3	1137.2	1102	A"
	777.1	781.5	779.8	767.3	780.3	781.4	764	A"
	168.3	156.7	157.2	158.7	159.1	154.2	150	A"
Cyclopentadiene	3283.2	3277.9	3276.6	3270.8	3269.5	3275.7	3091	A1
	3259.2	3254.4	3254.5	3247.1	3246.2	3253.3	3075	A1
	3078.3	3072.2	3070.7	3056.8	3065.3	3069.6	2886	A1
	1544.2	1531.8	1530.8	1520.4	1523.2	1528.0	1500	A1
	1419.4	1426.8	1424.5	1407.0	1429.2	1427.0	1378	A1
	1401.4	1408.0	1407.5	1389.0	1402.3	1409.3	1365	A1
	1120.7	1129.7	1129.6	1117.4	1133.8	1131.9	1106	A1

	1031.2	1028.6	1029.4	1024.4	1027.0	1028.5	994	A1
	944.1	942.6	943.8	936.4	940.0	943.1	915	A1
	799.8	804.5	808.2	796.1	805.8	808.3	802	A1
	1102.3	1111.7	1110.4	1096.7	1105.4	1113.1	1100	A2
	892.0	907.9	907.4	859.3	905.7	911.4	941	A2
	663.3	670.8	670.9	649.2	670.7	672.7	700	A2
	490.7	506.0	508.5	487.7	503.0	511.6	516	A2
	3130.2	3116.2	3115.8	3105.5	3109.2	3112.5	2900	B1
	908.9	926.7	931.8	889.8	928.7	934.8	925	B1
	892.5	920.7	927.6	878.0	919.8	932.6	891	B1
	670.4	685.5	688.3	654.9	686.8	691.2	664	B1
	325.4	337.1	337.1	327.0	336.3	340.0	350	B1
	3275.8	3271.3	3270.5	3263.7	3262.8	3269.6	3105	B2
	3250.3	3245.7	3245.9	3238.3	3237.0	3244.7	3043	B2
	1621.2	1613.3	1613.3	1593.1	1603.2	1611.3	1580	B2
	1323.4	1324.6	1325.2	1303.2	1316.6	1325.3	1292	B2
	1266.3	1272.5	1271.9	1251.4	1270.5	1273.6	1239	B2
	1117.6	1129.6	1128.6	1110.3	1123.0	1131.9	1090	B2
	987.0	988.0	988.1	976.2	986.2	988.3	959	B2
	805.8	810.7	814.9	801.0	812.7	814.9	805	B2
	3303.6	3289.7	3291.7	3288.9	3282.8	3287.8	3090	A'
	3205.7	3199.2	3198.3	3194.7	3189.3	3197.0	3015	A'
	3196.0	3189.3	3189.3	3183.4	3180.8	3187.6	3015	A'
	3193.5	3186.9	3187.1	3181.2	3178.3	3185.5	3015	A'
1,3-pentadiene	3187.7	3181.7	3179.5	3176.8	3169.6	3178.6	3015	A'
	3179.8	3165.2	3167.1	3156.1	3158.2	3162.9	2940	A'
	3073.6	3066.8	3067.7	3052.5	3060.8	3065.8	2919	A'
	1706.0	1696.4	1695.3	1686.9	1690.6	1693.2	1661	A'
	1651.0	1646.1	1645.5	1633.4	1639.7	1644.5	1610	A'

	1489.5	1507.8	1507.4	1485.2	1511.0	1512.0	1433	A'
	1452.9	1462.1	1460.5	1448.9	1458.0	1463.2	1422	A'
	1408.8	1415.6	1416.6	1396.1	1418.3	1418.0	1385	A'
	1321.7	1329.9	1331.3	1318.9	1327.1	1332.9	1316	A'
	1309.5	1315.3	1316.3	1305.6	1312.1	1317.4	1299	A'
	1284.9	1294.7	1296.6	1286.8	1293.0	1298.5	1280	A'
	1213.0	1214.5	1215.2	1207.7	1212.2	1215.3	1166	A'
	1117.9	1113.8	1114.5	1110.7	1112.5	1113.3	1037	A'
	1013.6	1023.8	1024.3	1002.1	1017.8	1026.7	985	A'
	949.5	959.5	960.5	940.1	949.5	962.7	862	A'
	480.7	481.0	482.8	479.3	480.0	482.4	483	A'
	448.2	448.0	450.2	449.5	446.6	449.6	418	A'
	202.9	205.6	207.9	209.9	207.1	207.9	212	A'
	3156.0	3138.0	3140.5	3129.2	3132.1	3135.4	2971	A"
	1476.4	1495.5	1494.6	1467.9	1498.5	1499.6	1453	A"
	1043.8	1058.3	1060.1	1038.4	1058.9	1063.1	1017	A"
	990.7	993.8	994.0	985.3	991.0	994.7	1002	A"
	912.3	913.5	914.0	906.2	911.0	914.1	912	A"
	855.6	887.6	894.3	872.7	887.7	900.3	899	A"
	791.1	813.5	819.1	797.3	811.2	823.0	820	A"
	624.1	635.9	637.1	625.2	631.5	639.7	615	A"
	130.5	132.7	133.0	134.8	131.2	133.5	129	A"

Table S4(b). Calculated harmonic vibrational frequencies for a selection of larger molecules using the Dunning correlation-consistent basis sets.

Mode symmetry	Full valence		Reduced active space		Experiment
A_g	3148.5	*(122.1)*	3234.2	*(207.8)*	3026.4
A_g	1686.3	*(63.4)*	1709.9	*(87.0)*	1622.9
A_g	1379.0	*(36.8)*	1404.0	*(61.8)*	1342.2
A_u	1037.2	*(14.2)*	1055.2	*(32.2)*	1023.0
B_{1u}	3131.2	*(142.5)*	3219.3	*(230.6)*	2988.7
B_{1u}	1491.1	*(47.6)*	1525.8	*(82.3)*	1443.5
B_{2g}	899.7	*(-39.9)*	888.1	*(-51.6)*	939.6
B_{2u}	3231.5	*(126.5)*	3328.0	*(223.0)*	3105.0
B_{2u}	834.5	*(8.5)*	851.2	*(25.2)*	826.0
B_{3g}	3208.0	*(122.0)*	3306.0	*(220.0)*	3086.0
B_{3g}	1245.9	*(28.8)*	1267.7	*(50.7)*	1217.0
B_{3u}	949.8	*(0.5)*	944.3	*(-5.0)*	949.3

Table S4(c). Calculated harmonic frequencies (cm^{-1}) using a full valence active space and a (2,2) active space for ethene with CASPT2 / 6-31G(d). Differences (cm^{-1}) from experiment are given in parentheses.

	Scaling factors	
Basis set	Full valence active space	Selected active space
6-31G(d)	1.010	0.946
6-31G(d,p)	0.988	0.936
6-31+G(d)	1.010	0.948
6-31+G(d,p)	0.986	0.942
6-31++G(d,p)	0.987	0.943
6-311G(d)	1.004	0.946
6-311G(d,p)	0.988	0.952
6-311+G(d)	1.002	0.955
6-311+G(d,p)	0.985	0.956
cc-pVDZ	1.001	0.955
cc-pVTZ	0.989	0.954
cc-pVQZ	0.984	0.955
aug-cc-pVDZ	1.001	0.960
aug-cc-pVTZ	0.990	0.957

Table S5. Scaling factors for the CASPT2 method recommended from this work.

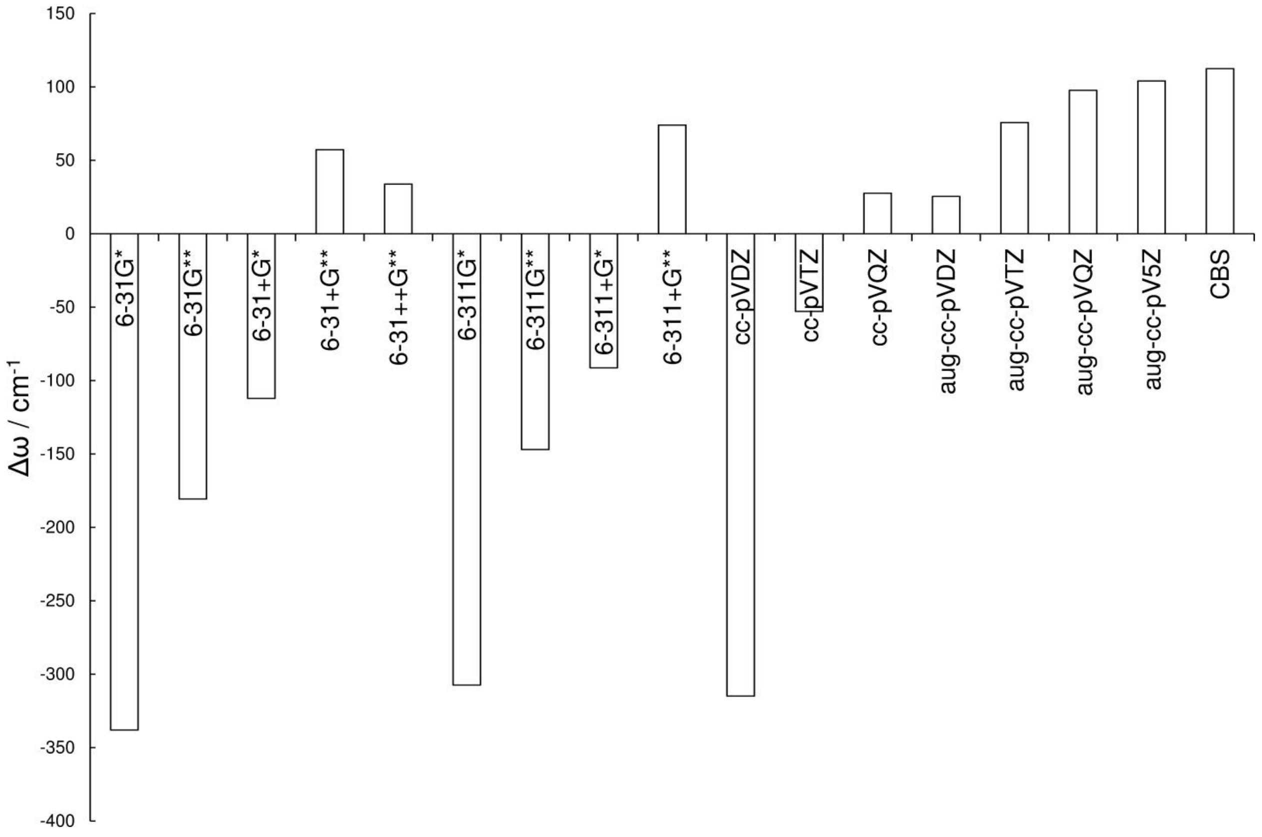

Figure S1. Difference from experiment of the harmonic frequency for OH^- with various basis sets.

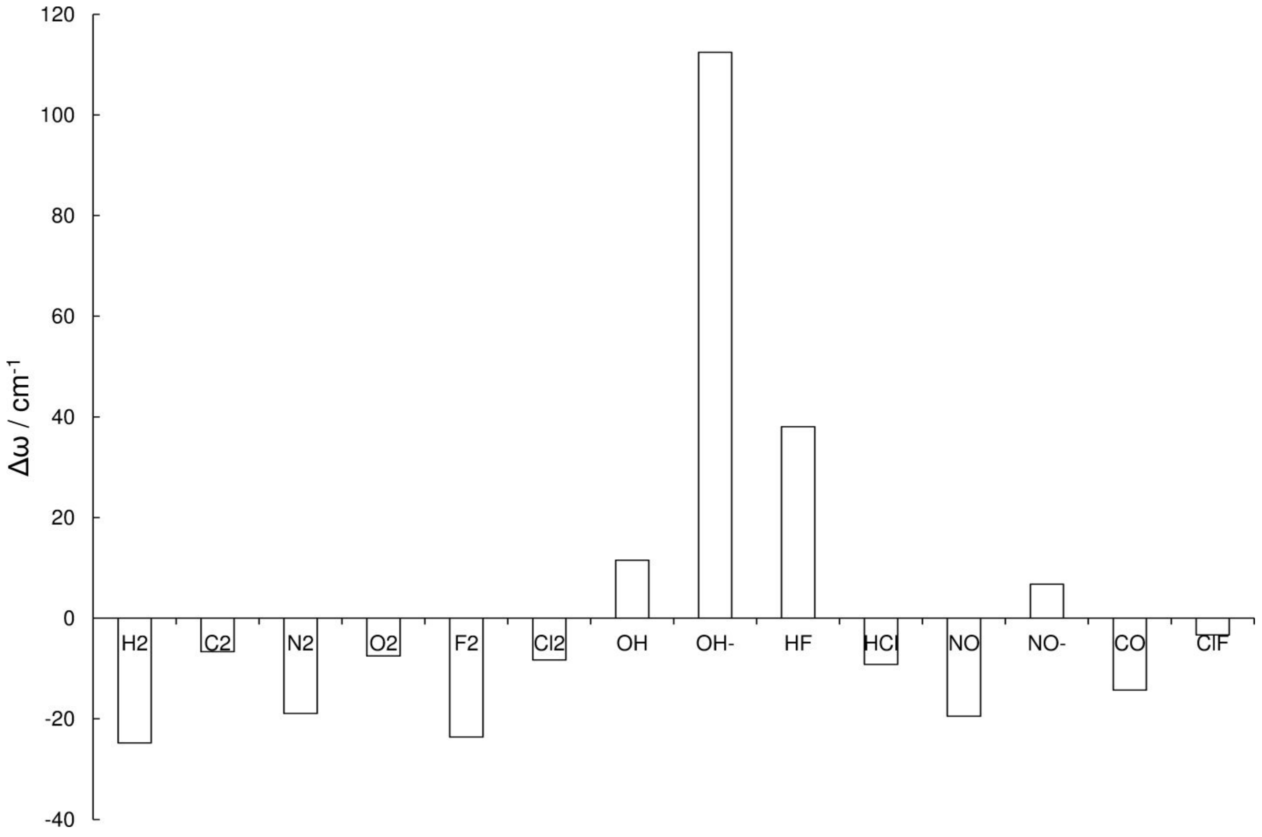

Figure S2. Difference between the calculated harmonic and experimental vibrational frequencies for the diatomic molecules considered (CASPT2 / CBS).

References

[1] NIST website: http://cccbdb.nist.gov/

[2] K. P. Huber and G. Herzberg, Molecular Spectra and Molecular Structure. IV. Constants of Diatomic Molecules; Van Nostrand Reinhold Co.: New York, 1979.

[3] K. K. Irikura, *J. Phys. Chem. Ref. Data* **36**, 389 (2007).

[4] T. Shimanouchi, *Nat. Stand. Ref. Data Ser., Nat. Bur. Stand. (U. S.)* **39**, 1 (1972).

[5] M. E. Jacox, *J. Phys. Chem. Ref. Data* **23**, 1 (1994).

[6] J. C. Owrutsky, N. H. Rosenbaum, L. M. Tack and R. J. Saykally, *J. Chem. Phys.* **83**, 5338 (1985).

[7] R. J. Le Roy, *Mol. Spect.* **194**, 189 (1999).

[8] NIST webbook, http://webbook.nist.gov/chemistry (accessed 27th June, 2017).

[9] K. Kim and W. T. King, *J. Chem. Phys.* **71**, 1967 (1979).

[10] L. V. Gurvich, I. V. Veyts and C. B. Alcock, Thermodynamic Properties of Individual Substances, Fouth Edition; Hemisphere Pub. Co.: New York, 1989.

[11] G. Herzberg, Electronic Spectra and Electronic Structure of Polyatomic Molecules; Van Nostrand: New York, 1966.

[12] T. Nakanaga, S. Kondo and S. Saeki, *J. Chem. Phys.* **76**, 3860 (1982).

[13] H. Hollenstien and Hs. H. Gunthard, *Spec. Acta A* **27A**, 2027 (1971).

[14] L. M. Sverdlov, M. A. Kovner and E. P. Krainov, Vibrational Spectra of Polyatomic Molecules; Wiley: New York, 1974.
